# Repeated HLA‐DRB1 and HLA‐DQB1 Mismatches Without Preformed DSA Affect Graft Survival, Rejection and DSA Development: A Multicenter Analysis

**DOI:** 10.1111/tan.70264

**Published:** 2025-05-19

**Authors:** D. A. J. van den Broek, S. Meziyerh, D. van der Helm, J. C. van den Born, J. S. F. Sanders, B. G. Hepkema, J. van de Wetering, J. A. Kal van Gestel, J. Kers, C. van Kooten, J. I. Rotmans, A. P. J. de Vries, D. L. Roelen

**Affiliations:** ^1^ Division of Nephrology, Department of Internal Medicine Leiden University Medical Center (LUMC), Leiden University Leiden the Netherlands; ^2^ Leiden Transplant Center Leiden University Medical Center (LUMC), Leiden University Leiden the Netherlands; ^3^ Department of Internal Medicine, Division of Nephrology University of Groningen, University Medical Center Groningen Groningen the Netherlands; ^4^ Transplantation Immunology, Department of Laboratory Medicine University Medical Center Groningen, University of Groningen Groningen the Netherlands; ^5^ Department of Internal Medicine, Erasmus Medical Center Transplant Institute University Medical Center Rotterdam the Netherlands; ^6^ Department of Pathology Leiden University Medical Center (LUMC) Leiden the Netherlands; ^7^ Department of Immunology, HLA Laboratory Leiden University Medical Center (LUMC) Leiden the Netherlands

**Keywords:** allograft survival, HLA, rejection, renal transplantation, repeated mismatch, risk‐stratification, sensitization

## Abstract

Repeat transplantations represent up to 20% of all kidney transplants. Whereas repeat transplantations in the presence of circulating donor‐specific HLA‐antibodies are generally avoided, the risk of repeated HLA‐mismatches (RMM) without detectable antibodies remains debated. This multicenter study evaluated the hazard of RMM, stratified by HLA‐class, on transplant outcomes in the absence of preformed donor‐specific antibodies. We included repeat kidney transplant recipients from January 2009 onward with available HLA typing for HLA‐A, ‐B, ‐C, ‐DRB1, ‐DRB3/4/5 and ‐DQB1 from current and previous donors. RMM were defined at the split serological antigen level, excluding patients with only HLA‐DP or HLA‐DRB3/4/5 RMM. Patients were included if: (a) preformed donor‐specific HLA‐antibodies (DSA) had never been detected and (b) this was confirmed by Luminex assays within 6 months pre‐transplantation. A competing risk model, adjusting for demographic factors and total HLA‐mismatch load while accounting for death as a competing risk, showed that HLA‐DRB1 and/or HLA‐DQB1 RMM significantly increased the risk of graft loss within 1 year post‐transplant (HR 3.75 [95% CI 1.51–9.34], *p* = 0.004). Cox proportional hazard models further linked these HLA‐class II RMM to higher risks of biopsy‐proven rejection (HR 1.98 [95% CI 1.04–3.76], *p* = 0.037) and DSA development (HR 9.89 [95% CI 1.92–50.99], *p* = 0.006), while no significant risks were observed for HLA‐class I RMM. Sensitivity analyses in patients screened for pretransplant DSA via single antigen bead assays, those with similar immunosuppression, and those with allelic RMM further confirmed these findings. These results suggest that avoiding HLA‐DRB1 and HLA‐DQB1 RMM, when feasible, may improve transplant outcomes.

**Abbreviations**
AMR: antibody‐mediated rejection
BPAR: biopsy proven acute rejection
BMI: body mass index
CDC: complement‐dependent cytotoxicity assay
dnDSA: de novo donor‐specific antibody
DSA: donor‐specific antibody
EMC: Erasmus Medical Center Rotterdam
HR: hazard ratio
IQR: interquartile range
LUMC: Leiden University Medical Center
RMM: repeated (HLA‐)mismatch
SAB: single antigen bead assay
TCMR: T‐cell mediated rejection
UMCG: University Medical Center Groningen

## 1 Introduction

Nearly 20% of patients on the Eurotransplant kidney transplant waitlist are listed for a repeat transplantation [1]. These candidates are often sensitised with antibodies to HLA, especially if their previous graft failed due to rejection [2, 3]. These preformed antibodies may increase the risk of (hyper)acute antibody‐mediated rejection (AMR) if the next allograft expresses a similar, so‐called repeated HLA‐mismatch (RMM) [4]. Although it is established that repeat transplantation in the presence of donor‐specific HLA‐antibodies (DSA) is associated with worse outcomes, it is debated whether this applies to repeat transplantation with grafts expressing RMM without detectable DSA. Older studies showed that repeated mismatches without detectable sensitisation increase the risk of rejection and graft loss, particularly in the context of HLA‐class II RMM [5, 6, 7]. However, these studies were conducted before the era of the Luminex single antigen bead (SAB) technology. A more recent collaborative transplant registry study found repeated HLA‐DR mismatches to be detrimental for transplant outcomes [8]. In contrast, a single‐centre study evaluated the impact of RMM without preformed DSA using the Luminex SAB platform and found no hazard [9]. A recent US registry study further alluded to this by only showing a significant hazard for HLA‐class II mismatches in patients with > 0% panel reactive antibodies (PRA) [10]. To contribute to the debate, we initiated a multicentre study to investigate the impact of HLA‐class I and class II RMM without detectable preformed DSA using the Luminex platform.

## 2 Methods and Materials

### 2.1 Patients

**FIGURE 1:**
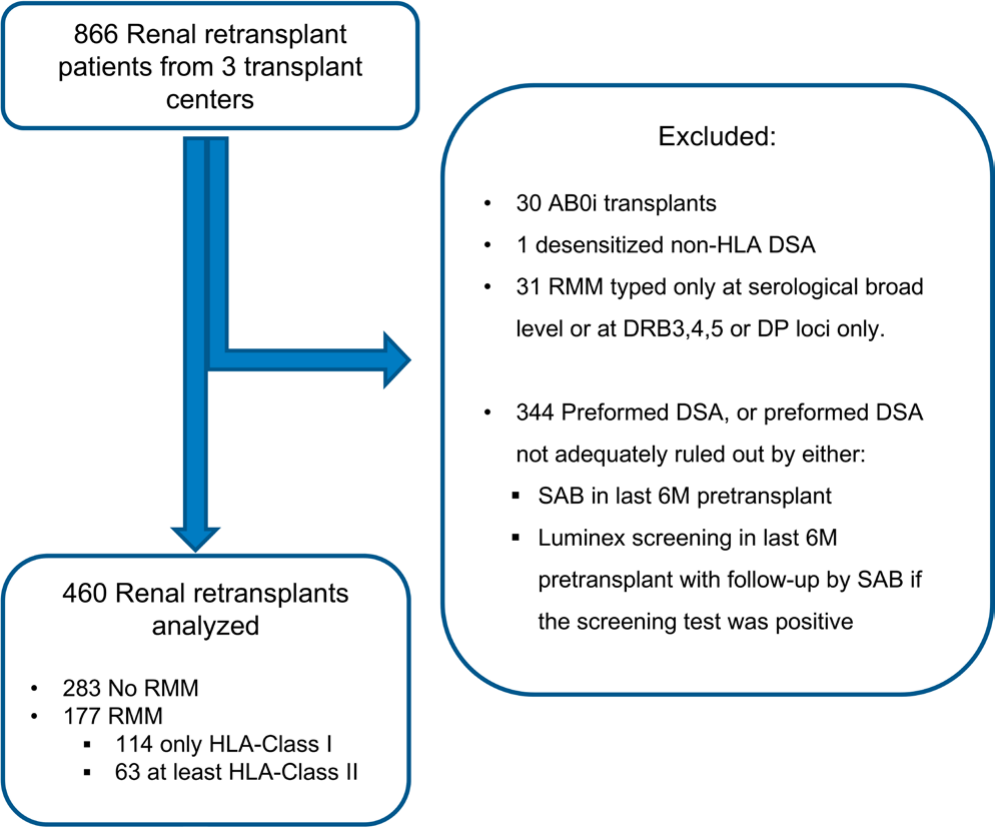
Patient selection flowchart. ABOi: ABO‐incompatible; DSA: donor‐specific antibodies; RMM: repeated (HLA)‐mismatch; SAB: single‐antigen bead.

We included patients who underwent a repeat kidney transplantation at Leiden University Medical Center (LUMC), Erasmus Medical Center (EMC), or University Medical Center Groningen (UMCG) from 2009 onwards, selecting those with complete HLA‐typing (HLA‐A, B, C, DRB1, DRB3/4/5, DQB1) for all previous donors. Of 866 identified, we excluded those with preformed DSA, ABO‐incompatibility, or preformed HLA‐DP antibodies if the current donor lacked HLA‐DP typing. Notably, patients with possible preformed DSA that could not be confirmed due to missing second‐field typing were excluded. Repeated mismatches were defined at the serological split antigen level as historic donors before 2009 were not HLA‐typed at second field, nor was material available. Patients were excluded if their RMM were defined only at the broad antigen level or if they had only repeated HLA‐DP or HLA‐DRB3/4/5 mismatches, given the significantly lower gene product expression of DRB3/4/5 compared to DRB1, which may affect pathogenicity [11, 12]. HLA‐DPB1 was excluded in the main analysis, as requiring complete HLA‐DPB1 typing for all historic donors and recipients would exclude > 90% of the cohort. Lastly, patients were only included if the absence of preformed DSA was confirmed within 6 months prior to transplant by Luminex single antigen bead (SAB) assays or Luminex screening assays followed by SAB if positive. This resulted in a final cohort of 460 patients for analysis. A detailed patient selection flowchart is provided in Figure 1. Institutional Review Board approval was obtained from all centers (protocol ID 132597). The clinical and research activities being reported are consistent with the Principles of the Declaration of Istanbul as outlined in the ‘Declaration of Istanbul on Organ Trafficking and Transplant Tourism’.

### 2.2 HLA‐Antibody Detection

All donor‐recipient pairs had negative unseparated T‐ and B‐cell complement‐dependent cytotoxicity crossmatches. T‐ and B‐cell flow‐cytometric crossmatching was performed routinely for living donor transplants only, and was negative for those donor‐recipient pairs. Per Eurotransplant regulations, all waitlisted transplant candidates were screened for HLA antibodies every 3 months.

At LUMC and EMC, HLA antibody testing was performed at the Leiden HLA Laboratory. Before 2012, One Lambda (One Lambda inc., Canoga Park, CA) Luminex screening and SAB kits were used; after 2012, Lifecodes kits (Werfen, Barcelona, Spain) were adopted. At UMCG, all HLA‐antibody screenings were conducted with Lifecodes assays. All assays followed the manufacturers' protocols. De novo (dn)DSA were assigned at second‐field resolution.

Routine dnDSA testing at LUMC occurred at 6 and 12 months post‐transplant, then annually and biennially after 4 years, or as clinically indicated (e.g., graft dysfunction or biopsy findings). At EMC, dnDSA assessment was performed only on indication. In this study, dnDSA data were available for LUMC and EMC only.

### 2.3 HLA‐Typing

At LUMC and EMC, recipient HLA typing was performed using Lifecodes Luminex kits (Lifecodes SSO, Werfen) until 2020, after which next‐generation sequencing (NGS) kits from GenDx (GenDx, Utrecht, The Netherlands) were used. At UMCG, Lifecodes kits were used for all recipient typings. Donors at all centres were typed with Olerup SSP kits (CareDx, Brisbane, CA).

### 2.4 Clinical Outcome Data

Demographic and clinical outcome data were obtained from transplant databases at each center. Only biopsy‐proven acute rejections (BPAR) were considered. At LUMC, all biopsies were retrospectively reassessed according to Banff 2022 criteria [13] by a single senior renal transplant pathologist (JK), who was blinded to RMM stratification but considered the clinical context.

### 2.5 Statistics

All statistical analyses were performed in R (R Foundation for Statistical Computing, Vienna, Austria). Categorical variables are presented as counts and percentages, while continuous variables are shown as medians with interquartile ranges (IQR). Patients were categorised into three groups based on RMM status: (1) Those without RMM; (2) Those with only HLA‐class I RMM; (3) Those with at least HLA‐class II RMM. Patients with simultaneous class I and class II RMM (*n* = 36) were included in this latter group due to sample sizes. Group comparisons were conducted using the Kruskal‐Wallis test for continuous variables and the Chi‐squared test for categorical variables. Kaplan–Meier analysis was used for time‐to‐event data, with group comparisons made using the Gehan‐Breslow‐Wilcoxon test. We hypothesised that RMM may trigger memory responses leading to early post‐transplant complications. Therefore, survival analyses were truncated at 1 year and 5 years post‐transplant. Full follow‐up data are provided in the Supporting Information. Multivariable analyses were performed using (a) Fine and Grey competing risk models for graft loss accounting for the competing risk of death [14], and (b) Cox proportional hazards models for rejection and DSA development. Models were adjusted for donor type and covariates that differed significantly between groups, including recipient age, donor age, transplant centre, PRA level and serological HLA‐mismatch load. A ratio of 10 events per covariate was maintained per analysis. Thus, fewer covariates were included depending on the event rate in some models. Missing data were limited for covariates (~ < 5%) and full‐case analyses were utilised.

### 2.6 Sensitivity Analyses

We conducted sensitivity analyses in an ‘allelic RMM sub‐cohort’, including only patients with ≥ 90% certainty that the serological RMM was also an allelic (second‐field) RMM based on regional HLA‐allele frequencies and known DRB1‐DQA1‐DQB1 linkage (details in Supporting Informations: Chapter II). Furthermore, we conducted sensitivity analyses for total biopsy‐proven rejection (TCMR Banff IA or higher and/or active, chronic active, or chronic AMR) using LUMC‐only patients, where all biopsies were reassessed according to Banff 2022 criteria. Lastly, sensitivity analyses were performed in patients with similar immunosuppression and in those screened for pretransplant DSA using only Luminex SAB.

## 3 Results

### 3.1 Patient Demographics

Patient demographics and baseline immunological characteristics are summarised in Table 1 and stratified by RMM HLA‐class. The study included 284 patients without RMM, 113 with only HLA‐class I RMM, 37 with only HLA‐class II RMM and 36 with both HLA‐class I and II RMM. The median follow‐up period was 4.15 years [IQR 1.77–6.89]. Given the limited patient count and the absence of significant outcome differences between the ‘only HLA‐class II RMM’ and ‘both HLA‐class I and II RMM’ groups (see below), we combined them as ‘HLA‐class II RMM’ for analyses. For clarity, we refer to ‘only HLA‐class I RMM’ as ‘HLA‐class I RMM’. Notably, no significant differences were found in recipient sex, BMI, donor type, number of previous transplants, time since last graft failure, induction agent, or immunosuppressive regimen across the three groups. However, patients with HLA‐class II RMM were older and had younger donors. RMM were more frequent in EMC compared to other centers. Significant differences were noted in PRA and serological HLA‐mismatch load.

**TABLE 1:** Baseline demographical and immunological factors, stratified for RMM per HLA‐class.

	No RMM (*n* = 284)	Class I RMM (*n* = 113)	Class II RMM (*n* = 63)	*p*
Sex				0.542
Male	173 (60.9%)	70 (61.9%)	34 (54.0%)	
Female	111 (39.1%)	43 (38.1%)	29 (46.0%)	
Recipient age at transplant	52.00 [41.00, 60.00]	54.00 [45.00, 63.00]	55.00 [44.50, 66.00]	**0.043**
Recipient BMI at transplant	24.52 [22.02, 27.62]	25.71 [22.86, 28.30]	25.60 [22.49, 29.65]	0.146
Number of previous renal transplants	1.00 [1.00, 1.00]	1.00 [1.00, 1.00]	1.00 [1.00, 1.00]	0.855
Time since last graft failure (years)	1.82 (0.38–5.69)	1.82 (0.62–3.27)	1.50 (0.42–2.90)	0.52
Donor age at transplant	53.00 [45.00, 59.00]	56.50 [46.00, 66.00]	51.00 [37.00, 60.00]	**0.034**
Donor type				0.235
Living	116 (40.8%)	52 (46.0%)	32 (50.8%)	
Deceased—donor after brain death	89 (31.3%)	33 (29.2%)	11 (17.5%)	
Deceased—donor after cardiac death	79 (27.8%)	28 (24.8%)	20 (31.7%)	
Induction agent				0.409
Alemtuzumab	14 (4.9%)	10 (8.8%)	3 (4.8%)	
IL2‐receptor antagonist	249 (87.7%)	102 (90.3%)	59 (93.7%)	
Thymoglobulin	5 (1.8%)	0 (0.0%)	1 (1.6%)	
Other	10 (3.6%)	1 (0.9%)	0 (0.0%)	
None	6 (2.1%)	0 (0.0%)	0 (0.0%)	
Immunosuppressive regime				0.400
Tac/MPA/CS	163 (57.4%)	68 (60.2%)	45 (71.4%)	
CSA/MPA/CS	19 (6.7%)	7 (6.2%)	0 (0.0%)	
Tac/mTORi/CS	14 (5.0%)	2 (1.8%)	1 (1.6%)	
Tac/AZA/CS	7 (2.5%)	6 (5.3%)	1 (1.5%)	
Tac/MPA	50 (17.6%)	19 (16.8%)	11 (17.5%)	
Belatacept based	5 (1.8%)	1 (0.9%)	0 (0.0%)	
Other	17 (5.8%)	7 (6.1%)	4 (6.4%)	
Unknown	9 (3.2%)	3 (2.7%)	1 (1.4%)	
Centre				**0.012**
LUMC	60 (21.1%)	26 (23.0%)	10 (15.9%)	
EMC	108 (38.0%)	58 (51.3%)	35 (55.6%)	
UMCG	116 (40.8%)	29 (25.7%)	18 (28.6%)	
PRA	14.0 [3.0–74.2]	5.00 [0.8–64.0]	5.00 [0.0–22.0]	**0.006**
Total HLA‐A mismatch	1.00 [0.00, 1.00]	1.00 [1.00, 2.00]	1.00 [1.00, 1.00]	**< 0.001**
Total HLA‐B mismatch	1.00 [1.00, 2.00]	1.00 [1.00, 2.00]	1.00 [1.00, 2.00]	**< 0.001**
Total HLA‐DRB1 mismatch	1.00 [0.00, 1.00]	1.00 [1.00, 1.00]	1.00 [1.00, 2.00]	**< 0.001**
Total HLA‐DQB1 mismatch	1.00 [0.00, 1.00]	1.00 [0.00, 1.00]	1.00 [1.00, 2.00]	**< 0.001**

*Note:* Detailed outline of baseline demographical and immunological factors per RMM HLA‐class stratum. Continuous values are displayed as median [IQR], categorical values as numbers (proportion). Statistical comparison for significant differences across groups is performed using Kruskal‐Wallis test for continuous variables and Chi‐squared test for categorical variables.

Abbreviations: AZA; Azathioprine; BMI: body‐mass index; CS: corticosteroids; CSA: cyclosporin A; EMC: Erasmus University Medical Centre; IL2: Interleukin‐2; IQR: interquartile range; LUMC: Leiden University Medical Centre; MPA: mycophenolate analogues; mTORi: mammalian target of rapamycin inhibitor; RMM: repeated HLA‐mismatch; Tac; tacrolimus; UMCG: University Medical Centre Groningen.

### 3.2 Graft Survival

**FIGURE 2:**
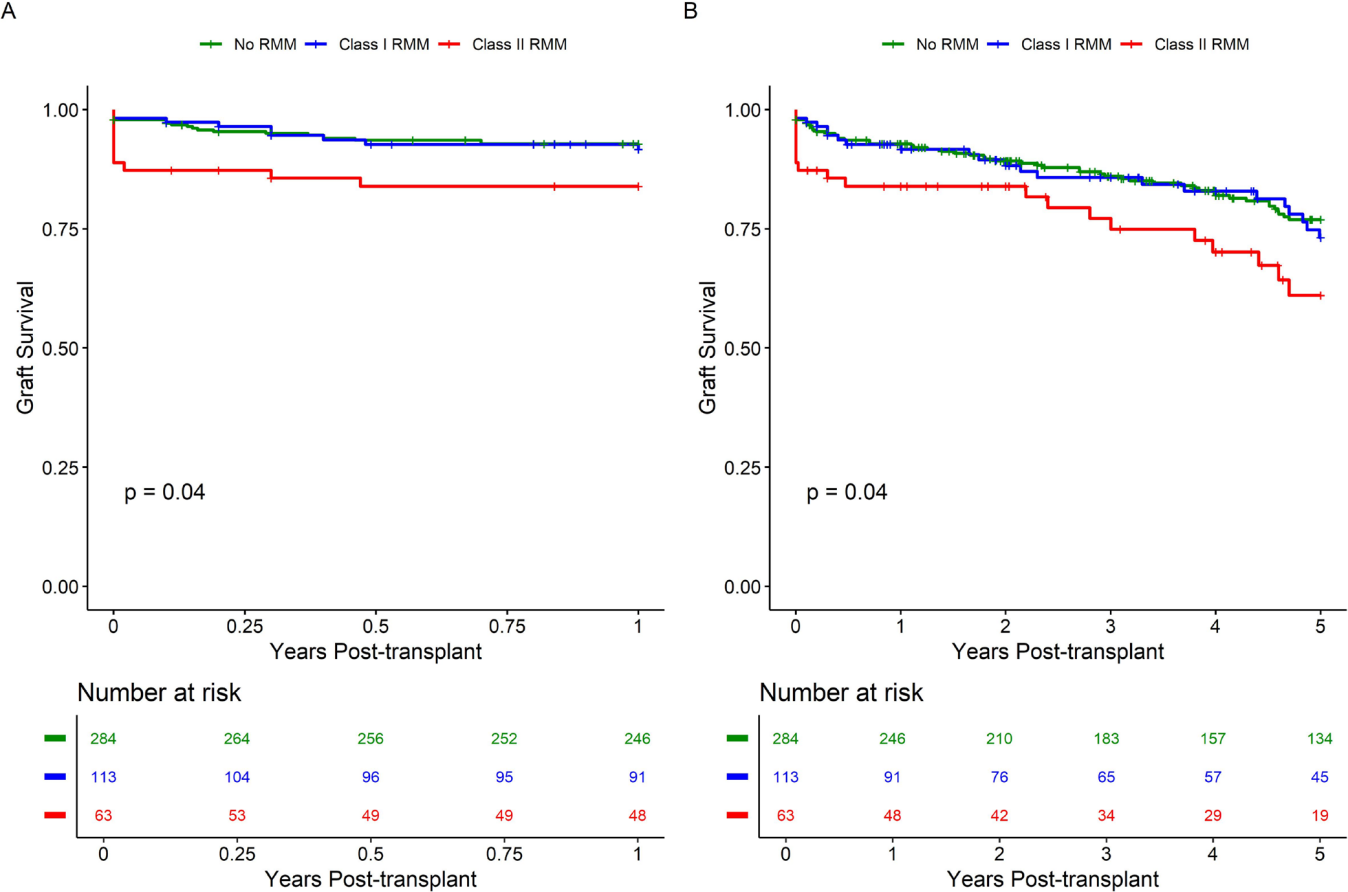
(A) Early graft survival truncated at one year post‐transplant. (B) Graft survival truncated at five years post‐transplant. All‐cause graft loss is defined by either return to dialysis, pre‐emptive retransplantation, or patient death. Statstical comparison between groups is performed using the Gehan‐Breslow‐Wilcoxon method. RMM: repeated HLA‐mismatch.

First, we assessed the effect of RMM HLA‐class on allograft survival. 1 and 5 year graft survival rates were 93% and 77% for patients without RMM; and 92% and 73% for those with HLA‐class I RMM. Graft survival was significantly worse for patients with HLA‐class II RMM at both 1 and 5 years post‐transplant compared to patients without RMM (84% and 61%; *p* = 0.040 and 0.041, respectively) (Figure 2). There was no significant difference in graft survival between all RMM patients and those without RMM (Figure S1).

Among first‐year graft losses, rejection accounted for 80% (8/10) in HLA‐class II RMM, 50% (10/20) in non‐RMM and 25% (2/8) in HLA‐class I RMM.

A Fine and Grey competing risk model, adjusting for recipient age, donor age, donor type, HLA‐mismatch load, transplant center, and PRA, confirmed a significant risk of graft loss at 5 years post‐transplant for HLA‐class II RMM patients (HR 3.96 [95% CI 1.86–8.45], *p* < 0.001) (Table 2). A model adjusting for donor type, donor age and HLA‐mismatch load confirmed a significant risk of graft loss at one‐year post‐transplant for such patients (HR 3.75 [95% CI 1.51–9.34], *p* = 0.004). No significant effects were observed for patients with HLA‐class I RMM on either one year or five‐years graft loss, compared to those without RMM.

**TABLE 2:** Multivariable Fine and Gray modelling for graft loss, accounting for the competing risk of death.

	One‐year graft loss			Five‐years graft loss		
Variable	Hazard ratio	95% CI	*p*	Hazard ratio	95% CI	*p*
No RMM	Ref	Ref	Ref	Ref	Ref	Ref
Class I RMM	0.85	0.30–2.45	0.77	1.36	0.68–2.72	0.39
Class II RMM	3.75	1.51–9.34	**0.004**	3.96	1.86–8.45	**< 0.001**
Recipient age	—	—	—	0.99	0.97–1.01	0.36
Donor age	1.02	0.98–1.05	0.20	1.02	0.998–1.050	0.07
Donor type: living	Ref	Ref	Ref	Ref	Ref	Ref
Donor type: deceased	6.09	1.83–20.3	**0.003**	3.15	1.57–6.36	**0.001**
Centre: EMC	—	—	—	Ref	Ref	Ref
Centre: LUMC	—	—	—	0.72	0.30–1.72	0.45
Centre: UMCG	—	—	—	0.81	0.40–1.64	0.56
PRA	—	—	—	1.006	0.994–1.010	0.14
Total HLA‐A/B/DR mismatch	0.84	0.66–1.09	0.21	0.86	0.70–1.06	0.17

*Note:* A multivariable Fine and Grey model to analyse the relationship between repeated mismatch HLA‐class with one‐ and five‐year graft loss, accounting for the competing risk of death. Associations are presented as hazard ratios with 95% confidence intervals and corresponding *p*‐values.

Abbreviations: EMC: Erasmus University Medical Centre; HLA: human leukocyte antigen; LUMC: Leiden University Medical Centre; PRA: panel reactive antibodies RMM: repeated HLA‐mismatch; UMCG: University Medical Centre Groningen.

Notably, comparison between ‘only HLA‐class II RMM’ and ‘both HLA‐class I and II RMM’ showed no significant differences at one‐year post‐transplant (HR 0.64 [95% CI 0.16–2.64], *p* = 0.50) or five‐years post‐transplant (HR 1.23 [95% CI 0.41–3.66], *p* = 0.72).

### 3.3 Sensitivity Analyses

A sensitivity analysis in patients without preformed DSA as screened with the Luminex SAB assay confirmed a significant risk for graft loss in patients with HLA‐class II RMM at both one‐year post‐transplant (HR 8.37 [95% CI 2.73–25.64], *p* = 0.002) and five‐years post‐transplant (HR 4.62 [95% CI 1.51–14.13], *p* = 0.007), using the same models as for the main analysis.

An alternative sensitivity analysis among patients with similar immunosuppressive protocols, receiving IL2‐receptor antagonist induction with tacrolimus, mycophenolates and corticosteroids maintenance (*n* = 243) confirmed significant hazards for graft loss in patients with HLA‐class II RMM at one‐year post‐transplant (HR 6.15 [95% CI 2.07–18.31], *p* = 0.001) and five‐years post‐transplant (HR 3.95 [95% CI 1.48–10.52], *p* = 0.006). A sub‐cohort analysis focusing on allelic repeated mismatches also demonstrated a significant risk of graft loss at one‐year post‐transplant (HR 4.14 [95% CI 1.26–13.60], *p* = 0.019) and five‐years post‐transplant (HR 4.90 [95% CI 1.74–13.79], *p* = 0.008) for HLA‐class II RMM patients. No significant effect of class I RMM was observed in this sub‐cohort.

### 3.4 Rejection

#### 3.4.1 Total Biopsy‐Proven Rejections

**FIGURE 3:**
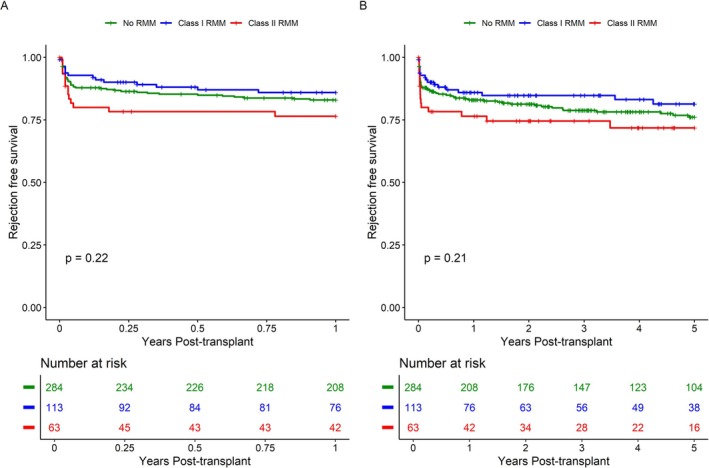
Rejection‐free survival, stratified for repeated mismatch per HLA‐class. (A) Early biopsy proven rejection‐free survival truncated at one‐year post‐transplant. (B) Biopsy proven rejection‐free survival truncated at five‐years post‐transplant. Statistical comparison between groups is performed using the Gehan‐Breslow‐Wilcoxon method. RMM: repeated HLA‐mismatch.

Next, we assessed the effects of RMM HLA‐class on biopsy‐proven rejection rates. Rejection‐free survival at one‐ and five‐years post‐transplant was 83% and 76% for patients without RMM; 86% and 81% for those with class I RMM; and 77% and 72% for those with class II RMM. No significant differences were observed at either timepoint (*p* = 0.21 and *p* = 0.20, respectively). (Figure 3).

Importantly, multivariable analysis revealed a significant risk of early biopsy‐proven rejection at 1 year for HLA‐class II RMM (HR 1.98 [95% CI 1.04–3.76], *p* = 0.037) (Table 3), after correcting for the considerable baseline demographical differences. This remained a non‐significant trend at 5 years (HR 1.60 [95% CI 0.86–2.96], *p* = 0.14). No association was found between HLA‐class I RMM and rejection.

**TABLE 3:** Multivariable Cox proportional hazard modelling of biopsy‐proven rejection.

	One‐year biopsy proven rejections			Five‐years biopsy proven rejections		
Variable	Hazard ratio	Hazard ratio	Hazard ratio	Hazard ratio	95% CI	*p*
No RMM	Ref	Ref	Ref	Ref	Ref	Ref
Class I RMM	0.79	0.43–1.42	0.43	0.69	0.40–1.20	0.19
Class II RMM	1.98	1.04–3.76	**0.037**	1.60	0.86–2.96	0.14
Recipient age	0.98	0.962–0.995	**0.01**	0.98	0.96–0.99	**0.004**
Donor age	1.03	1.01–1.05	**0.008**	1.03	1.01–1.05	**0.003**
Donor type: living	Ref	Ref	Ref	Ref	Ref	Ref
Donor type: deceased	1.37	0.83–2.27	0.22	1.31	0.83–2.05	0.25
Centre: EMC	Ref	Ref	Ref	Ref	Ref	Ref
Centre: LUMC	0.96	0.54–1.72	0.89	1.14	0.68–1.89	0.62
Centre: UMCG	0.47	0.26–0.86	**0.013**	0.53	0.31–0.91	**0.02**
PRA	1.01	0.999–1.012	0.08	1.007	1.001–1.013	**0.04**
Total HLA‐A/B/DR mismatch	—	—	—	1.13	0.97–1.31	0.11

*Note:* A multivariable Cox proportional hazard model to analyse the relationship between repeated mismatch HLA‐class and one‐ and five‐years biopsy proven rejections. Associations are presented as hazard ratios with 95% confidence intervals and corresponding *p*‐values.

Abbreviations: EMC: Erasmus University Medical Centre; HLA: human leukocyte antigen; LUMC: Leiden University Medical Centre; PRA: panel reactive antibodies RMM: repeated HLA‐mismatch; UMCG: University Medical Centre Groningen.

### 3.5 Sensitivity Analyses

In a sensitivity analysis of LUMC patients, where biopsies were reassessed per Banff' 22 criteria, HLA‐class II RMM was significantly associated with total biopsy‐proven rejection at one‐year post‐transplant (HR 5.38 [95% CI 1.22–23.78], *p* = 0.026) and five‐years post‐transplant (HR 4.99 [95% CI 1.16–21.38], *p* = 0.030), using the same models as for the main analysis. The separate analysis for AMR and TCMR in the Leiden cohort is found in the Supporting Informations: Chapter III. No significant effect of repeated mismatch HLA‐class was observed for either AMR (Figure S4) or TCMR (Figure S5), though a non‐significant trend towards increased risk of AMR was noted for patients with HLA‐class II RMM.

When assessing LUMC patients in our allelic RMM sub‐cohort, class II RMM remained significantly associated with biopsy‐proven rejection at five‐years post‐transplant (HR 5.00 [95% CI 1.03–24.12], *p* = 0.04), compared to those without RMM. This remained a strong yet non‐significant trend at one‐year post‐transplant (HR 4.63 [95% CI 0.90–23.92], *p* = 0.07). HLA‐Class I allelic RMM were not associated with rejection at either timepoint.

### 3.6 DSA Development

**FIGURE 4:**
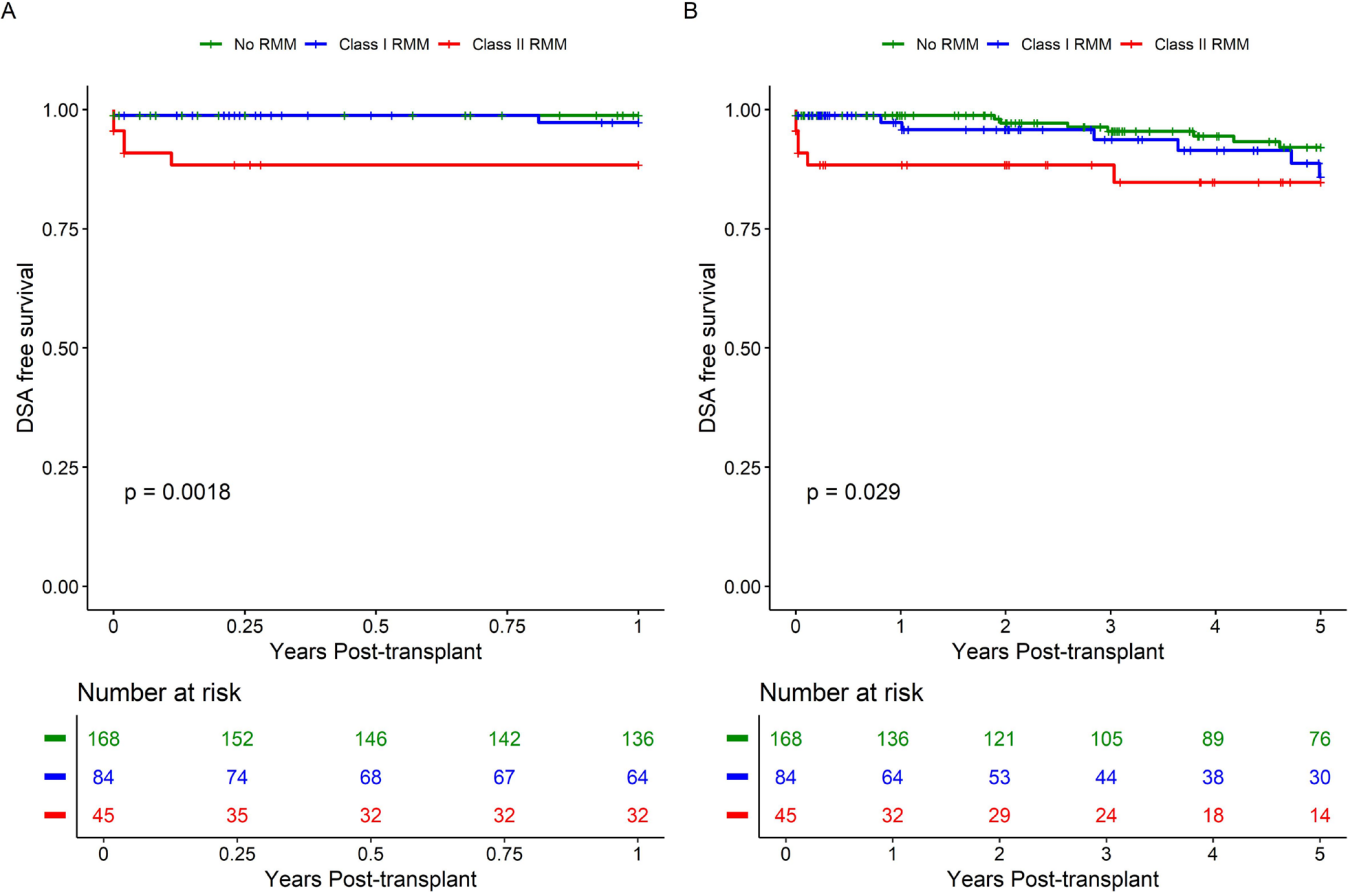
DSA‐free survival, stratified for repeated mismatch per HLA‐class. Post‐transplant DSA‐free survival for the LUMC and EMC centres. (A) DSA‐free survival truncated at one‐year post‐transplant. (B) DSA‐free survival truncated at five‐years post‐transplant. Statistical comparison between groups is performed using the Gehan‐Breslow‐Wilcoxon method. RMM: repeated HLA‐mismatch.

Subsequently, we evaluated the effect of RMM HLA‐class on the risk of developing DSA post‐transplant. One‐ and five‐year DSA‐free survival rates were 99% and 92% for those without RMM, 96% and 86% for those with class I RMM, and 88% and 84% for those with class II RMM. Patients with HLA‐class II RMM had a significantly higher risk to develop DSA within both the first‐year post‐transplant (*p* = 0.002) and within five‐years post‐transplant (*p* = 0.018) (Figure 4). The respective univariate hazard ratio at one‐ and five‐years post‐transplant for DSA development compared to non‐RMM was 2.00 ([95% CI 0.28–14.17], *p* = 0.49) and 1.70 ([95% CI 0.63–4.58], *p* = 0.29) for class I RMM; and 9.89 ([95% CI 1.92–50.99], *p* = 0.006) and 2.86 ([95% CI 1.01–8.03], *p* = 0.047) for class II RMM. A multivariable analysis correcting for transplant center and HLA‐mismatch load confirms a significant risk of DSA development at five‐years post‐transplant for patients with class II RMM (HR 2.99 [95% CI 1.00–8.91], *p* = 0.049) (Table 4). The event rate of DSA development within the first year did not allow for a meaningful multivariable analysis within this timeframe.

**TABLE 4:** Multivariable Cox proportional hazard modelling of DSA development.

	Five‐years DSA development		
Variable	Hazard ratio	95% CI	*p*
No RMM	Ref	Ref	Ref
Class I RMM	1.61	0.58–4.43	0.36
Class II RMM	2.99	1.00–8.91	**0.049**
Centre: EMC	Ref	Ref	Ref
Centre: LUMC	20.1	0.86–4.69	0.11
Total HLA‐A/B/DR mismatch	1.08	0.81–1.44	0.62

*Note:* A multivariable Cox proportional hazard model to analyse the relationship between repeated mismatch HLA‐class and DSA development at five‐years post‐transplant, adjusted for transplant center and total HLA‐mismatch load. Associations are presented as hazard ratios with 95% confidence intervals and corresponding *p*‐values.

Abbreviations: CI: confidence interval; EMC: Erasmus University Medical Centre; HLA: human leukocyte antigen; LUMC: Leiden University Medical Centre; RMM: repeated mismatch.

### 3.7 Sensitivity Analyses

A sensitivity analysis in patients screened for pretransplant DSA using Luminex SAB confirmed a significant risk for DSA development at one‐year post‐transplant (HR 13.42 [95% CI 1.50–120.30], *p* = 0.02). In this cohort, the repeated mismatch was the target in 75% of DSA‐positive patients with HLA‐class II RMM. A sensitivity analysis in patients included in our allelic RMM sub‐cohort maintained a significant hazard for DSA development in patients with class II RMM within the first year (HR 9.89 [95% CI 1.92–50.99], *p* = 0.006), where the RMM was the target in all DSA‐positive patients with HLA‐class II RMM. A sensitivity analysis in patients with similar immunosuppressive protocols, receiving IL2‐receptor antagonist induction with tacrolimus, mycophenolates and corticosteroids maintenance confirmed a significant hazard for DSA development in patients with class II RMM within the first year (HR 5.71 [95% CI 1.05–31.20], *p* = 0.04). Lastly, a sensitivity analysis in only LUMC patients, where assessment of DSA is prospectively conducted, again found a significant hazard for class II RMM, compared to those without RMM (HR 12.19 [95% CI 1.11–134.51], *p* = 0.04).

## 4 Discussion

This is the first multi‐center study to date to examine the impact of repeated HLA‐mismatches without preformed DSA, as determined by Luminex technology, in patients with contemporary immunosuppression. There is scant evidence on the impact of repeated mismatches without preformed DSA in kidney transplantation, and this research aims to refine the understanding of risks specifically associated with HLA‐class II RMM.

Our real‐world findings demonstrate that repeated HLA‐DRB1 and HLA‐DQB1 mismatches without preformed DSA significantly reduced graft survival and increased the risk of biopsy‐proven rejection and DSA development. These associations remained significant after adjusting for demographic factors and total HLA mismatch load. Furthermore, sensitivity analyses confirmed the robustness of these findings in patients with comparable immunosuppressive protocols and those screened for preformed DSA using specifically Luminex SAB assays. The latter of which is important as undetected preformed DSA, especially low‐grade HLA‐class II antibodies, may be missed by Luminex screening assays [15]. Crucially, despite not having all historical donors HLA‐typed at second‐field resolution, the impact and effect sizes of RMM remained materially unchanged in our sensitivity analyses of patients included in our allelic repeated mismatch sub‐cohort, in which the involved serological mismatch was approximated as an allelic (second‐field) repeated mismatch with > 90% certainty based on known allelic frequencies or linkage of DRB1, DQA1 and DQB1 in our population. This argues against the hypothesis that the absence of a significant effect of HLA‐class I RMM was a result of patients not being re‐exposed to the exact same molecular mismatch. It also reinforces that the observed significant effects of HLA‐class II RMM were not diminished by this factor.

The higher rate of graft loss for patients with HLA‐class II RMM in our study is likely driven by the increased risk of rejection, which appears antibody‐mediated, considering the observed greater risk of early DSA development targeting the RMM in the large majority of cases and the trend toward AMR in the LUMC sub‐cohort. Furthermore, the clear association of HLA‐class II RMM with early biopsy‐proven rejections and the fact that nearly all first‐year graft losses in patients with HLA‐class II RMM were attributed to rejection further highlight the clinical significance of these repeated mismatches and the potential memory response they may have triggered.

Unfortunately, not all biopsies in this cohort were Banff classified, which might have provided greater insight into the histopathological subtype of rejection. Yet, as highlighted in a recent review, this binary classification does not fully capture the heterogeneity of rejection [16]. Thus, while our study was unable to definitively distinguish between AMR and TCMR, the key finding remains that HLA‐class II RMM are significantly associated with biopsy‐proven rejection and adverse graft outcomes. Considerable baseline differences in patient demographics (recipient age, donor age, transplant centre, vPRA) may have influenced the univariate analysis for BPAR. However, after adjusting for these covariates in the multivariable analysis, HLA‐class II RMM remained a significant risk factor for early BPAR, underscoring the robustness and clinical relevance of the multivariable findings over the univariate results.

Our results are in line with the prior recent European registry study by Pipeleers et al. [8], yet contrast the US registry data by Tinckham et al. [10]. We attribute this discrepancy primarily to differences in DSA assessment methodologies. The US study conducted in patients transplanted before 2011 relied largely on complement‐dependent cytotoxicity (CDC) crossmatching rather than more sensitive Luminex‐based assays. We also contrast the single‐center study by Lucisano et al. [9] This may owe to our larger sample size, pooled data from three centers, and stratification by HLA class. Notably, when patients were simply categorised as having any RMM (regardless of class), no significant difference was observed, aligning with Lucisano et al. [9] This suggests that the specific impact of HLA class II RMM may be masked in broader categorizations.

A key question arising from our findings is why HLA‐DRB1 and DQB1 RMM have a greater impact on outcomes than HLA‐class I RMM. Although 50% of patients with HLA‐class II RMM also had HLA‐class I RMM, outcomes did not differ between those with and without additional HLA‐class I RMM, and HLA‐class I RMM alone did not significantly affect transplant outcomes. This suggests that HLA‐class II RMM confer an independent risk, even in the absence of detectable circulating DSA.

HLA‐class II antibodies, particularly DQ‐antibodies, are the most common DSA, indicating a greater predisposition to class II humoral sensitization [2]. This may also indicate a heightened likelihood of developing HLA‐class II‐specific memory B‐cells capable of initiating AMR via a memory response [17, 18]. Notably, HLA‐specific memory B‐cells can persist without detectable circulating HLA‐antibodies [19, 20].

Beyond humoral sensitisation, T‐cell memory may also contribute to the detrimental effects of class II RMM. CD4^+^ memory T‐cells may activate CD8^+^ cytotoxic T‐cells [21], leading to cellular infiltrates and TCMR [22, 23], ultimately affecting graft survival [24]. However, we noted no effect of RMM on TCMR‐free survival, suggesting a stronger role for B‐cell mechanisms, though our sample size to detect Banff classified TCMR and AMR was too limited. Still, experimental models indicate that donor‐reactive CD4+ memory T‐cells can drive AMR through interactions with the humoral immune response [25, 26], warranting further investigation into the role of donor‐specific T‐cell memory.

Various working groups on sensitisation in transplantation have also acknowledged the risk posed by cellular memory in sensitised patients [27, 28]. Our findings of reduced graft survival, combined with an increased risk of early DSA development and rejection in patients with class II RMM, may underscore the clinical significance of these observations.

A crucial clinical question is whether repeated HLA‐mismatches should be avoided in renal retransplant candidates. We detected no significant effects on any transplant outcome of HLA‐class I RMM, perhaps indicating they are less relevant in a cohort of our size. For HLA‐class II RMM, our analysis focused on the combined impact of HLA‐DRB1 and HLA‐DQB1, excluding HLA‐DPB1 and HLA‐DRB3/4/5 due to the study criteria. Separate analyses of patients with RMM at these loci were not possible due to the sample size of those subpopulations.

For repeated HLA‐DRB1 and HLA‐DQB1 mismatches, we suggest that the balance between the increased time on dialysis, rejection risk and allocation probability should guide decisions on assigning unacceptable antigens. Avoiding these class II RMM may be advisable for retransplant candidates with low calculated PRA, as it likely will not significantly impact their waiting time due to sufficient acceptable antigens. Candidates for live donations with low PRA could be coached into paired donor exchange programmes or might have other better HLA‐matched donors available.

However, for highly sensitised patients, excluding all DRB1 and DQB1 RMM from consideration may severely limit donor availability. Alternative strategies, such as modified induction therapy, warrant exploration, though evidence on their effectiveness in RMM patients without preformed DSA remains limited. Future clinical strategies may also include pre‐transplant assessment of HLA‐specific B‐ or T‐cell memory to refine risk stratification [29]. However, such assays are not yet widely available, and memory B‐cell testing may yield false negatives since memory B‐cells tend to reside in lymphoid organs rather than circulate [29, 30, 31].

We acknowledge several limitations in our study. First, although all patients included in our cohort were confirmed to have no detectable DSA within 6 months prior to transplantation and historically, we cannot definitively exclude the possibility of newly formed DSA emerging in the per Eurotransplant mandatory three‐month interval between the last antibody assessment and the transplant itself. This limitation of incipient sensitisation within mandatory screening intervals (e.g., after reduction of residual immunosuppression) reflects current clinical practice, where logistical constraints often preclude Luminex‐based DSA testing on the day of transplantation, particularly in the deceased donor setting. Nonetheless, cell‐based crossmatches (CDC for deceased donor transplants and flow crossmatch for all living donor transplants) were negative for all included patients, though this does not rule out lower strength DSA. Second, RMM were identified based on serological equivalents due to the lack of second‐field resolution for many historical donor HLA‐typings. However, results in our allelic RMM sub‐cohort reflected those of the total cohort, supporting the validity of our findings. We also could not assess the impact of HLA‐DP or HLA‐DRB3/4/5 RMM due to limited data. Additionally, the sample sizes and the typing limitations precluded analysis of shared epitope effects. Future studies could further explore whether molecular similarities between historical and current mismatches influence rejection and graft loss risk. However, as this effect may vary by HLA‐locus [32], a substantially larger cohort than ours will likely be required to draw definitive conclusions. Lastly, DSA screening strategies varied between centres; while LUMC conducted routine post‐transplant DSA screening, EMC only performed DSA testing when clinically indicated. Nonetheless, within the LUMC cohort, class II RMM remained a significant risk factor for DSA development, underscoring our findings.

Importantly, despite factors like rejection classifications and DSA screening strategy potentially affecting specific data interpretation, none of these limitations or arguments apply to graft survival. This outcome is objective and robust, and the significant negative association of HLA‐DRB1 and HLA‐DQB1 RMM with both early and longer‐term graft survival remains undeniable in our view.

In conclusion, repeated mismatches at HLA‐DRB1 and HLA‐DQB1, even in the absence of detectable preformed DSA, are associated with an increased risk of graft loss in renal retransplant patients. This risk appears primarily driven by a higher incidence of rejection and DSA development. These findings support considering avoidance of HLA‐DRB1 and HLA‐DQB1 RMM in retransplant candidates where feasible, balancing the potential benefits against the limitations imposed on organ allocation. Further research is necessary to clarify the risks associated with class I RMM, as well as with HLA‐DRB3/4/5, and HLA‐DP repeated mismatches.

## Conflicts of Interest

The authors declare no conflicts of interest.

## Supporting information


Data S1.

## Data Availability

Due to the sensitive nature of (genetic) HLA typing data upon which the main stratification of patients within this study is based, data cannot be publicly shared. Certain types of data may be shared for research purposes upon request to the corresponding author.
