# Supplementary material for: Repeated HLA‐DRB1 and HLA‐DQB1 Mismatches Without Preformed DSA Affect Graft Survival, Rejection and DSA Development: A Multicenter Analysis
Source: HLA. 2025 May 19;105(5):e70264. doi: 10.1111/tan.70264 (PMC12087429; doi:10.1111/tan.70264)
Supplement: Supplementary file 1 — Data S1. [file TAN-105-e70264-s001.docx]

**Supplementary materials**

Table of Contents

**Chapter I: STROBE statement2**

**Chapter II: Supplementary methods5**

**Attribution of allelic repeated mismatches**5

**Supplemental Table** **1**8

**Supplemental Table** **2**14

**Chapter III: Supplementary Results**15

**Supplemental Figure 1**15

**Supplemental Figure 2**16

**Supplemental Figure 3**17

**Antibody-mediated rejection**18

**T-cell mediated rejection**18

**Supplemental Figure 4**19

**Supplemental Figure 5**20

**Supplemental Figure 6**21

**Chapter IV: Supplementary References**21

**Chapter I: STROBE Statement—checklist of items that should be included in reports of observational studies**

|  | Item No | Recommendation | Pages |
| --- | --- | --- | --- |
| **Title and abstract** | 1 | (*a*) Indicate the study’s design with a commonly used term in the title or the abstract | 1-2 |
|  |  | (*b*) Provide in the abstract an informative and balanced summary of what was done and what was found | 1-2 |
| Introduction | | |  |
| Background/rationale | 2 | Explain the scientific background and rationale for the investigation being reported | 3 |
| Objectives | 3 | State specific objectives, including any prespecified hypotheses | 3-4 |
| Methods | | |  |
| Study design | 4 | Present key elements of study design early in the paper | 3-4 |
| Setting | 5 | Describe the setting, locations, and relevant dates, including periods of recruitment, exposure, follow-up, and data collection | 3-5 |
| Participants | 6 | (*a*) *Cohort study*—Give the eligibility criteria, and the sources and methods of selection of participants. Describe methods of follow-up  *Case-control study*—Give the eligibility criteria, and the sources and methods of case ascertainment and control selection. Give the rationale for the choice of cases and controls  *Cross-sectional study*—Give the eligibility criteria, and the sources and methods of selection of participants | 3-5 |
|  |  | (*b*) *Cohort study*—For matched studies, give matching criteria and number of exposed and unexposed  *Case-control study*—For matched studies, give matching criteria and the number of controls per case |  |
| Variables | 7 | Clearly define all outcomes, exposures, predictors, potential confounders, and effect modifiers. Give diagnostic criteria, if applicable | 5-7 |
| Data sources/ measurement | 8* | For each variable of interest, give sources of data and details of methods of assessment (measurement). Describe comparability of assessment methods if there is more than one group | 4-7 |
| Bias | 9 | Describe any efforts to address potential sources of bias | 6-7 |
| Study size | 10 | Explain how the study size was arrived at | 3-4 |
| Quantitative variables | 11 | Explain how quantitative variables were handled in the analyses. If applicable, describe which groupings were chosen and why | 6 |
| Statistical methods | 12 | (*a*) Describe all statistical methods, including those used to control for confounding | 6-7 |
|  |  | (*b*) Describe any methods used to examine subgroups and interactions | 6-7 |
|  |  | (*c*) Explain how missing data were addressed | 6 |
|  |  | (*d*) *Cohort study*—If applicable, explain how loss to follow-up was addressed  *Case-control study*—If applicable, explain how matching of cases and controls was addressed  *Cross-sectional study*—If applicable, describe analytical methods taking account of sampling strategy | 6 |
|  |  | (*e*) Describe any sensitivity analyses | 6-7 |

| Results | | |  |
| --- | --- | --- | --- |
| Participants | 13* | (a) Report numbers of individuals at each stage of study—eg numbers potentially eligible, examined for eligibility, confirmed eligible, included in the study, completing follow-up, and analysed | 7 |
|  |  | (b) Give reasons for non-participation at each stage | 7 |
|  |  | (c) Consider use of a flow diagram | 7, Figure 1 |
| Descriptive data | 14* | (a) Give characteristics of study participants (eg demographic, clinical, social) and information on exposures and potential confounders | 7, Table 1 |
|  |  | (b) Indicate number of participants with missing data for each variable of interest |  |
|  |  | (c) *Cohort study*—Summarise follow-up time (eg, average and total amount) |  |
| Outcome data | 15* | *Cohort study*—Report numbers of outcome events or summary measures over time | *9-10, 13, 15-16 figures 2-4* |
|  |  | *Case-control study—*Report numbers in each exposure category, or summary measures of exposure |  |
|  |  | *Cross-sectional study—*Report numbers of outcome events or summary measures |  |
| Main results | 16 | (*a*) Give unadjusted estimates and, if applicable, confounder-adjusted estimates and their precision (eg, 95% confidence interval). Make clear which confounders were adjusted for and why they were included | *9-10, 13, 15-16*, Tables 2-4 |
|  |  | (*b*) Report category boundaries when continuous variables were categorized |  |
|  |  | (*c*) If relevant, consider translating estimates of relative risk into absolute risk for a meaningful time period |  |
| Other analyses | 17 | Report other analyses done—eg analyses of subgroups and interactions, and sensitivity analyses | 10, 13, 16 |
| Discussion | | |  |
| Key results | 18 | Summarise key results with reference to study objectives | 18-19 |
| Limitations | 19 | Discuss limitations of the study, taking into account sources of potential bias or imprecision. Discuss both direction and magnitude of any potential bias | 21-22 |
| Interpretation | 20 | Give a cautious overall interpretation of results considering objectives, limitations, multiplicity of analyses, results from similar studies, and other relevant evidence | 19-21 |
| Generalisability | 21 | Discuss the generalisability (external validity) of the study results | 21-22 |
| Other information | | |  |
| Funding | 22 | Give the source of funding and the role of the funders for the present study and, if applicable, for the original study on which the present article is based | 23 |

*Give information separately for cases and controls in case-control studies and, if applicable, for exposed and unexposed groups in cohort and cross-sectional studies.

**Note:** An Explanation and Elaboration article discusses each checklist item and gives methodological background and published examples of transparent reporting. The STROBE checklist is best used in conjunction with this article (freely available on the Web sites of PLoS Medicine at http://www.plosmedicine.org/, Annals of Internal Medicine at http://www.annals.org/, and Epidemiology at http://www.epidem.com/). Information on the STROBE Initiative is available at www.strobe-statement.org.

**Chapter II: Supplementary methods**
**Attribution of allelic repeated mismatches.**

We performed sensitivity analyses in patients where the serological RMM was known or highly (>=90%) likely to be an allelic, second-field, RMM, to ensure that the effect of repeated HLA-mismatches is not misinterpreted due to the fact that not all serological repeated mismatches are molecular repeated mismatches. We thereby estimated the likelihood that a serological mismatch was also an allelic repeated mismatch based on allele frequencies within serological antigen groups. While many alleles exist for each serological antigen, only a few are commonly observed, particularly in the Dutch (predominantly Caucasian) population. For instance, the list of known HLA-A1 alleles represents alleles ranging from A*01:01 to A*01:478. However, in our population, 99.7% of serological HLA-A1 is A*01:01, with the remaining 0.3% A*01:03. Therefore, the likelihood that any random repeated serological HLA-A1 mismatch is also an allelic repeated mismatch is 0.997^2^ + 0.003^2^ = 0.9936 or 99.36%.
To conduct this analysis, we determined allele frequencies for all observed serological RMM within a cohort of 844 renal transplant recipients (transplanted after 2016 in Leiden) and their donors, all typed at second-field resolution. In addition, we determined the relative frequency of HLA-alleles in two separate Dutch cohorts that are available on the online database allelefrequencies.net^1^, which are both of similar size as our own typed cohort. These cohorts were a sampling of random blood donors in 2008 in Leiden and a sampling of registered bone marrow donors in 2010 in Germany, who had identified themselves to be of Dutch descent. The mean allele frequency across these three datasets was used to estimate allelic repeated mismatch probability (Supplemental Table 1). For patients that had more than one HLA-class I RMM, the likelihood that at least one of these is also an allelic repeated mismatch is greater than their sole respective probabilities. For instance, the likelihood that any random serological HLA-B13 repeated mismatch is also an allelic repeated mismatch is 86%. This is 72% for any random serological HLA-B62 repeated mismatch. If a patient receives a retransplant with both a HLA-B13 and a HLA-B62 repeated mismatch, the likelihood that at least one of these is also an allelic repeated mismatch can be rewritten as:
1 – (likelihood that neither are a RMM), or: 1 – (0.14^2^ + 0.28^2^) = 0.902. In this case, the likelihood that this patient received an allelic class I RMM is 90,2%. In general terms this can be written as:

$$P_{RMM\_allelic} =1-\prod_{i=1}^{N} (1-\sum_{j} p_{i,j}^{2})$$

Where ***i*** represents the index serological antigen, ***j*** represents the index allele within serological antigen ***i,*** ***p_i,j_*** represents the frequency of the *j*-th allele within the *i*-th serological group and ***N*** represents the number of serological repeated mismatches.
We applied a cutoff of >=90% probability for inclusion as a class I allelic RMM recipient, excluding patients below this threshold.
For HLA-class II RMM, molecular repeated mismatch probabilities per antigen were generally lower except for DR7, DR9, DR17, and DQ8 due to higher allelic diversity. Therefore, we leveraged known **HLA-DR/DQ haplotype linkages** in our cohort to infer whether serological RMM were also allelic repeated mismatches. (Supplemental Table 2)
As an example: a patient is considered whose previous donor had the following serological DR and DQ typings: DR1, DR7, DQ2, DQ5 and the current donor has the serological DR and DQ typings: DR10, DR17, DQ2, DQ5. Assume the patient was fully mismatched with both donors. Serologically, the repeated mismatches are DQ2 and DQ5.
By assessing the linkage of DR and DQ, it can be deduced/attributed that the previous donor’s haplotypes likely were:
DR1 – DQ5 and DR7 – DQ2 and the current donor’s haplotypes likely were: DR10 – DQ5 and DR17 – DQ2. This is because DR1 and DR10 are not known to be linked to DQ2, but are known to be linked to DQ5. Whereas DR7 and DR17 are not known to be associated with DQ5, but are known to be associated with DQ2 instead. This is also observed in our typed cohort. (Supplemental table 2)
DR1 and DR10 are known to only be linked to DQA1*01:01P/DQB1*05:01P and not any other DQ5 heterodimer in our population.
However the DQ2 linked to DR7 is different than the DQ2 linked to DR17, as they are respectively DQA1*02:01P/DQB1*02:01P and DQA1*05:01P/DQB1*02:01P.
Therefore, the DQ5 is very likely an allelic repeated mismatch, but the DQ2 is not.
By utilizing this knowledge of linkage, we attributed the repeated class II serological mismatch in 33 of 63 patients as a repeated allelic mismatch. In 13 of 63 patients the serological repeated mismatch was attributed as molecularly different and in 17 of 63 patients it could not be certainly attributed.
The above process led to a sub-cohort of patients with strong likelihood (>=90%) of allelic repeated mismatches, totaling 91 patients with only class I allelic repeated mismatches with a median probability of an allelic repeated mismatch of 96.3% (IQR 91.0% - 99.4%), 33 patients with at least a class II allelic repeated mismatch based on linkage and 284 patients without repeated mismatch.

**Supplemental table 1**

| **RMM antigen** | **Corresponding Allele** | **Frequency in Leiden >2016 cohort** | **Frequency in Leiden blood donors** | **Frequency in DKMS Netherlands** | **Mean frequency** | **Probability of molecular RMM** |
| --- | --- | --- | --- | --- | --- | --- |
| A1 | A*01:01 | 0.993 | 1.000 | 0.998 | 0.997 | 0.99 |
| A1 | A*01:03 | 0.007 | 0.000 | 0.002 | 0.003 |  |
| A2 | A*02:01 | 0.926 | 0.961 | 0.970 | 0.952 | 0.91 |
| A2 | A*02:02 | 0.011 | 0.000 | 0.001 | 0.004 |  |
| A2 | A*02:03 | 0.007 | 0.003 | 0.000 | 0.003 |  |
| A2 | A*02:05 | 0.020 | 0.016 | 0.017 | 0.018 |  |
| A2 | A*02:06 | 0.012 | 0.010 | 0.007 | 0.010 |  |
| A2 | A*02:07 | 0.004 | 0.000 | 0.000 | 0.001 |  |
| A2 | A*02:09 | 0.001 | 0.000 | 0.000 | 0.000 |  |
| A2 | A*02:10 | 0.001 | 0.000 | 0.000 | 0.000 |  |
| A2 | A*02:11 | 0.013 | 0.000 | 0.000 | 0.004 |  |
| A2 | A*02:17 | 0.002 | 0.000 | 0.002 | 0.001 |  |
| A2 | A*02:20 | 0.000 | 0.010 | 0.001 | 0.004 |  |
| A2 | A*02:22 | 0.001 | 0.000 | 0.000 | 0.000 |  |
| A2 | A*02:35 | 0.000 | 0.000 | 0.001 | 0.000 |  |
| A2 | A*02:39 | 0.002 | 0.000 | 0.000 | 0.001 |  |
| A3 | A*03:01 | 0.962 | 0.980 | 0.979 | 0.974 | 0.95 |
| A3 | A*03:02 | 0.038 | 0.020 | 0.021 | 0.026 |  |
| A24 | A*24:02 | 0.919 | 0.957 | 0.975 | 0.951 | 0.91 |
| A24 | A*24:03 | 0.027 | 0.021 | 0.013 | 0.021 |  |
| A24 | A*24:07 | 0.057 | 0.021 | 0.007 | 0.028 |  |
| A24 | A*24:13 | 0.000 | 0.000 | 0.004 | 0.001 |  |
| A24 | A*24:58 | 0.003 | 0.000 | 0.000 | 0.001 |  |
| A24 | A*24:72 | 0.003 | 0.000 | 0.000 | 0.001 |  |
| A31 | A*31:01 | 0.961 | 1.000 | 1.000 | 0.987 | 0.97 |
| A31 | A*31:04 | 0.013 | 0.000 | 0.000 | 0.004 |  |
| A31 | A*31:06 | 0.026 | 0.000 | 0.000 | 0.009 |  |
| A26 | A*26:01 | 0.905 | 0.992 | 0.963 | 0.953 | 0.91 |
| A26 | A*26:08 | 0.041 | 0.008 | 0.019 | 0.023 |  |
| A26 | A*26:09 | 0.000 | 0.000 | 0.019 | 0.006 |  |
| A26 | A*26:15 | 0.054 | 0.000 | 0.000 | 0.018 |  |
| A11 | A*11:01 | 0.982 | 1.000 | 1.000 | 0.994 | 0.99 |
| A11 | A*11:02 | 0.009 | 0.000 | 0.000 | 0.003 |  |
| A11 | A*11:03 | 0.009 | 0.000 | 0.000 | 0.003 |  |
| A68 | A*68:01 | 0.819 | 0.860 | 0.864 | 0.848 | 0.74 |
| A68 | A*68:02 | 0.181 | 0.140 | 0.136 | 0.152 |  |
| A29 | A*29:01 | 0.090 | 0.074 | 0.081 | 0.082 | 0.83 |
| A29 | A*29:02 | 0.872 | 0.926 | 0.919 | 0.906 |  |
| A29 | A*29:10 | 0.026 | 0.000 | 0.000 | 0.009 |  |
| A29 | A*29:95 | 0.013 | 0.000 | 0.000 | 0.004 |  |
|  |  |  |  |  |  |  |
| A30 | A*30:01 | 0.557 | 0.619 | 0.658 | 0.611 | 0.53 |
| A30 | A*30:02 | 0.481 | 0.381 | 0.321 | 0.394 |  |
| A30 | A*30:04 | 0.063 | 0.000 | 0.021 | 0.028 |  |
| A31 | A*31:01 | 1.000 | 1.000 | 1.000 | 1.000 | 1.00 |
| A32 | A*32:01 | 1.000 | 1.000 | 0.989 | 0.996 | 0.99 |
| A32 | A*32:04 | 0.000 | 0.000 | 0.011 | 0.004 |  |
| B7 | B*07:02 | 0.957 | 0.974 | 0.968 | 0.966 | 0.93 |
| B7 | B*07:03 | 0.000 | 0.006 | 0.003 | 0.003 |  |
| B7 | B*07:04 | 0.002 | 0.000 | 0.005 | 0.003 |  |
| B7 | B*07:05 | 0.017 | 0.019 | 0.021 | 0.019 |  |
| B7 | B*07:06 | 0.024 | 0.000 | 0.000 | 0.008 |  |
| B7 | B*07:14 | 0.000 | 0.000 | 0.003 | 0.001 |  |
| B8 | B*08:01 | 0.997 | 1.000 | 1.000 | 0.999 | 1.00 |
| B8 | B*08:09 | 0.003 | 0.000 | 0.000 | 0.001 |  |
| B13 | B*13:01 | 0.138 | 0.074 | 0.017 | 0.076 | 0.86 |
| B13 | B*13:02 | 0.863 | 0.926 | 0.983 | 0.924 |  |
| B18 | B*18:01 | 0.952 | 1.000 | 0.981 | 0.978 | 0.96 |
| B18 | B*18:02 | 0.007 | 0.000 | 0.000 | 0.002 |  |
| B18 | B*18:03 | 0.027 | 0.000 | 0.009 | 0.012 |  |
| B18 | B*18:04 | 0.007 | 0.000 | 0.000 | 0.002 |  |
| B18 | B*18:05 | 0.007 | 0.000 | 0.000 | 0.002 |  |
| B18 | B*18:09 | 0.000 | 0.000 | 0.009 | 0.003 |  |
| B35 | B*35:01 | 0.636 | 0.759 | 0.722 | 0.706 | 0.56 |
| B35 | B*35:02 | 0.088 | 0.057 | 0.073 | 0.073 |  |
| B35 | B*35:03 | 0.188 | 0.149 | 0.158 | 0.165 |  |
| B35 | B*35:04 | 0.000 | 0.000 | 0.004 | 0.001 |  |
| B35 | B*35:05 | 0.023 | 0.011 | 0.004 | 0.013 |  |
| B35 | B*35:06 | 0.000 | 0.000 | 0.004 | 0.001 |  |
| B35 | B*35:08 | 0.047 | 0.023 | 0.035 | 0.035 |  |
| B35 | B*35:09 | 0.003 | 0.000 | 0.000 | 0.001 |  |
| B35 | B*35:11 | 0.003 | 0.000 | 0.000 | 0.001 |  |
| B35 | B*35:30 | 0.003 | 0.000 | 0.000 | 0.001 |  |
| B35 | B*35:43 | 0.003 | 0.000 | 0.000 | 0.001 |  |
| B38 | B*38:01 | 0.893 | 1.000 | 1.000 | 0.964 | 0.93 |
| B38 | B*38:02 | 0.093 | 0.000 | 0.000 | 0.031 |  |
| B38 | B*38:16 | 0.013 | 0.000 | 0.000 | 0.004 |  |
| B39 | B*39:01 | 0.691 | 0.667 | 0.762 | 0.707 | 0.56 |
| B39 | B*39:06 | 0.182 | 0.333 | 0.238 | 0.251 |  |
| B39 | B*39:09 | 0.018 | 0.000 | 0.000 | 0.006 |  |
| B39 | B*39:10 | 0.091 | 0.000 | 0.000 | 0.030 |  |
| B39 | B*39:31 | 0.018 | 0.000 | 0.000 | 0.006 |  |
| B44 | B*44:02 | 0.606 | 0.641 | 0.653 | 0.633 | 0.51 |
| B44 | B*44:03 | 0.388 | 0.308 | 0.284 | 0.327 |  |
| B44 | B*44:04 | 0.011 | 0.017 | 0.006 | 0.011 |  |
| B44 | B*44:05 | 0.036 | 0.034 | 0.057 | 0.042 |  |
| B44 | B*44:15 | 0.003 | 0.000 | 0.000 | 0.001 |  |
| B44 | B*44:27 | 0.011 | 0.000 | 0.000 | 0.004 |  |
| B51 | B*51:01 | 0.917 | 0.982 | 0.972 | 0.957 | 0.92 |
| B51 | B*51:02 | 0.021 | 0.000 | 0.008 | 0.009 |  |
| B51 | B*51:05 | 0.005 | 0.000 | 0.000 | 0.002 |  |
| B51 | B*51:07 | 0.005 | 0.000 | 0.021 | 0.009 |  |
| B51 | B*51:08 | 0.031 | 0.018 | 0.000 | 0.016 |  |
| B51 | B*51:19 | 0.005 | 0.000 | 0.000 | 0.002 |  |
| B51 | B*51:20 | 0.005 | 0.000 | 0.000 | 0.002 |  |
| B51 | B*51:50 | 0.010 | 0.000 | 0.000 | 0.003 |  |
| B53 | B*53:01 | 1.000 | 1.000 | 1.000 | 1.000 | 1.00 |
| B55 | B*55:01 | 1.000 | 1.000 | 1.000 | 1.000 | 1.00 |
| B60 | B*40:01 | 0.995 | 1.000 | 1.000 | 0.998 | 1.00 |
| B60 | B*40:10 | 0.005 | 0.000 | 0.000 | 0.002 |  |
| B61 | B*40:02 | 0.674 | 0.846 | 0.980 | 0.833 | 0.72 |
| B61 | B*40:04 | 0.011 | 0.000 | 0.000 | 0.004 |  |
| B61 | B*40:06 | 0.304 | 0.154 | 0.020 | 0.159 |  |
| B61 | B*40:16 | 0.011 | 0.000 | 0.000 | 0.004 |  |
| B62 | B*15:01 | 0.955 | 0.963 | 0.972 | 0.963 | 0.93 |
| B62 | B*15:06 | 0.004 | 0.000 | 0.000 | 0.001 |  |
| B62 | B*15:07 | 0.016 | 0.038 | 0.016 | 0.023 |  |
| B62 | B*15:24 | 0.012 | 0.000 | 0.008 | 0.007 |  |
| B62 | B*15:25 | 0.004 | 0.000 | 0.000 | 0.001 |  |
| B62 | B*15:27 | 0.004 | 0.000 | 0.000 | 0.001 |  |
| B62 | B*15:39 | 0.004 | 0.000 | 0.000 | 0.001 |  |
| B62 | B*15:56 | 0.000 | 0.000 | 0.004 | 0.001 |  |
| B65 | B*14:02 | 1.000 | 1.000 | 1.000 | 1.000 | 1.00 |
| C1 | C*01:02 | 1.000 | 1.000 | 0.987 | 0.996 | 0.99 |
| C1 | C*01:32 | 0.000 | 0.000 | 0.013 | 0.004 |  |
| C2 | C*02:02 | 0.917 | 0.977 | 0.959 | 0.951 | 0.91 |
| C2 | C*02:06 | 0.000 | 0.000 | 0.007 | 0.002 |  |
| C2 | C*02:10 | 0.083 | 0.023 | 0.034 | 0.047 |  |
| C4 | C*04:01 | 0.934 | 0.981 | 0.997 | 0.970 | 0.94 |
| C4 | C*04:03 | 0.049 | 0.010 | 0.000 | 0.020 |  |
| C4 | C*04:04 | 0.002 | 0.000 | 0.000 | 0.001 |  |
| C4 | C*04:06 | 0.002 | 0.000 | 0.000 | 0.001 |  |
| C4 | C*04:07 | 0.002 | 0.000 | 0.000 | 0.001 |  |
| C4 | C*04:09N | 0.007 | 0.010 | 0.000 | 0.006 |  |
| C4 | C*04:10 | 0.002 | 0.000 | 0.000 | 0.001 |  |
| C4 | C*04:15 | 0.000 | 0.000 | 0.003 | 0.001 |  |
| C4 | C*04:33 | 0.002 | 0.000 | 0.000 | 0.001 |  |
| C4 | C*04:82 | 0.002 | 0.000 | 0.000 | 0.001 |  |
| C5 | C*05:01 | 0.996 | 1.000 | 1.000 | 0.999 | 1.00 |
| C5 | C*05:03 | 0.004 | 0.000 | 0.000 | 0.001 |  |
| C6 | C*06:02 | 0.996 | 1.000 | 0.978 | 0.991 | 0.98 |
| C6 | C*06:08 | 0.004 | 0.000 | 0.000 | 0.001 |  |
| C6 | C*06:17 | 0.000 | 0.000 | 0.014 | 0.005 |  |
| C6 | C*06:53 | 0.000 | 0.000 | 0.008 | 0.003 |  |
| C7 | C*07:01 | 0.488 | 0.462 | 0.483 | 0.477 | 0.47 |
| C7 | C*07:02 | 0.509 | 0.494 | 0.456 | 0.487 |  |
| C7 | C*07:04 | 0.051 | 0.038 | 0.060 | 0.050 |  |
| C7 | C*07:06 | 0.014 | 0.000 | 0.000 | 0.005 |  |
| C7 | C*07:18 | 0.015 | 0.006 | 0.000 | 0.007 |  |
| C7 | C*07:25 | 0.001 | 0.000 | 0.000 | 0.000 |  |
| C7 | C*07:35 | 0.001 | 0.000 | 0.000 | 0.000 |  |
| C7 | C*07:109 | 0.000 | 0.000 | 0.001 | 0.000 |  |
| C8 | C*08:01 | 0.291 | 0.091 | 0.075 | 0.152 | 0.73 |
| C8 | C*08:02 | 0.686 | 0.909 | 0.925 | 0.840 |  |
| C8 | C*08:03 | 0.023 | 0.000 | 0.000 | 0.008 |  |
| C9 | C*03:03 | 1.000 | 1.000 | 1.000 | 1.000 | 1.00 |
| C10 | C*03:02 | 0.081 | 0.027 | 0.025 | 0.044 | 0.92 |
| C10 | C*03:04 | 0.919 | 0.973 | 0.975 | 0.956 |  |
| C12 | C*12:02 | 0.245 | 0.140 | 0.128 | 0.171 | 0.72 |
| C12 | C*12:03 | 0.755 | 0.860 | 0.872 | 0.829 |  |
| C16 | C*16:01 | 0.786 | 0.900 | 0.916 | 0.868 | 0.76 |
| C16 | C*16:02 | 0.175 | 0.007 | 0.033 | 0.071 |  |
| C16 | C*16:04 | 0.029 | 0.003 | 0.051 | 0.028 |  |
| DR1 | DRB1*01:01 | 0.881 | 0.899 | 0.903 | 0.895 | 0.81 |
| DR1 | DRB1*01:02 | 0.068 | 0.067 | 0.056 | 0.064 |  |
| DR1 | DRB1*01:03 | 0.051 | 0.034 | 0.035 | 0.040 |  |
| DR1 | DRB1*01:07 | 0.000 | 0.000 | 0.006 | 0.002 |  |
| DR4 | DRB1*04:01 | 0.503 | 0.595 | 0.599 | 0.566 | 0.38 |
| DR4 | DRB1*04:02 | 0.055 | 0.025 | 0.029 | 0.036 |  |
| DR4 | DRB1*04:03 | 0.053 | 0.049 | 0.055 | 0.052 |  |
| DR4 | DRB1*04:04 | 0.206 | 0.233 | 0.206 | 0.215 |  |
| DR4 | DRB1*04:05 | 0.059 | 0.018 | 0.031 | 0.036 |  |
| DR4 | DRB1*04:06 | 0.008 | 0.000 | 0.000 | 0.003 |  |
| DR4 | DRB1*04:07 | 0.067 | 0.055 | 0.064 | 0.062 |  |
| DR4 | DRB1*04:08 | 0.025 | 0.025 | 0.000 | 0.017 |  |
| DR4 | DRB1*04:14 | 0.000 | 0.000 | 0.003 | 0.001 |  |
| DR4 | DRB1*04:15 | 0.002 | 0.000 | 0.000 | 0.001 |  |
| DR7 | DRB1*07:01 | 1.000 | 1.000 | 1.000 | 1.000 | 1.00 |
| DR9 | DRB1*09:01 | 1.000 | 1.000 | 1.000 | 1.000 | 1.00 |
| DR11 | DRB1*11:01 | 0.670 | 0.725 | 0.684 | 0.693 | 0.54 |
| DR11 | DRB1*11:02 | 0.054 | 0.013 | 0.007 | 0.024 |  |
| DR11 | DRB1*11:03 | 0.027 | 0.038 | 0.066 | 0.044 |  |
| DR11 | DRB1*11:04 | 0.235 | 0.225 | 0.227 | 0.229 |  |
| DR11 | DRB1*11:06 | 0.006 | 0.000 | 0.000 | 0.002 |  |
| DR11 | DRB1*11:12 | 0.006 | 0.000 | 0.000 | 0.002 |  |
| DR11 | DRB1*11:14 | 0.000 | 0.000 | 0.015 | 0.005 |  |
| DR11 | DRB1*11:35 | 0.003 | 0.000 | 0.000 | 0.001 |  |
| DR12 | DRB1*12:01 | 0.593 | 0.889 | 0.886 | 0.789 | 0.67 |
| DR12 | DRB1*12:02 | 0.407 | 0.111 | 0.114 | 0.211 |  |
| DR13 | DRB1*13:01 | 0.475 | 0.525 | 0.547 | 0.516 | 0.44 |
| DR13 | DRB1*13:02 | 0.420 | 0.407 | 0.397 | 0.408 |  |
| DR13 | DRB1*13:03 | 0.085 | 0.059 | 0.051 | 0.065 |  |
| DR13 | DRB1*13:04 | 0.004 | 0.000 | 0.000 | 0.001 |  |
| DR13 | DRB1*13:05 | 0.007 | 0.008 | 0.005 | 0.007 |  |
| DR13 | DRB1*13:10 | 0.004 | 0.000 | 0.000 | 0.001 |  |
| DR13 | DRB1*13:14 | 0.002 | 0.000 | 0.000 | 0.001 |  |
| DR14 | DRB1*14:01P | 0.789 | 0.951 | 0.959 | 0.900 | 0.81 |
| DR14 | DRB1*14:02 | 0.007 | 0.000 | 0.000 | 0.002 |  |
| DR14 | DRB1*14:04 | 0.156 | 0.019 | 0.014 | 0.063 |  |
| DR14 | DRB1*14:05 | 0.007 | 0.000 | 0.000 | 0.002 |  |
| DR14 | DRB1*14:06 | 0.000 | 0.011 | 0.000 | 0.004 |  |
| DR14 | DRB1*14:07 | 0.000 | 0.019 | 0.027 | 0.015 |  |
| DR14 | DRB1*14:08 | 0.007 | 0.000 | 0.000 | 0.002 |  |
| DR14 | DRB1*14:15 | 0.007 | 0.000 | 0.000 | 0.002 |  |
| DR14 | DRB1*14:21 | 0.007 | 0.000 | 0.000 | 0.002 |  |
| DR14 | DRB1*14:24 | 0.007 | 0.000 | 0.000 | 0.002 |  |
| DR15 | DRB1*15:01 | 0.843 | 0.931 | 0.960 | 0.911 | 0.84 |
| DR15 | DRB1*15:02 | 0.106 | 0.063 | 0.034 | 0.067 |  |
| DR15 | DRB1*15:03 | 0.033 | 0.006 | 0.003 | 0.014 |  |
| DR15 | DRB1*15:04 | 0.004 | 0.000 | 0.000 | 0.001 |  |
| DR15 | DRB1*15:06 | 0.006 | 0.000 | 0.000 | 0.002 |  |
| DR15 | DRB1*15:18 | 0.000 | 0.000 | 0.003 | 0.001 |  |
| DR15 | DRB1*15:20 | 0.044 | 0.000 | 0.000 | 0.015 |  |
| DR17 | DRB1*03:01 | 1.000 | 1.000 | 1.000 | 1.000 | 1.00 |
| DQ2 | DQA1*02:01P/DQB1*02:01P | 0.453 |  |  | 0.453 | 0.47 |
| DQ2 | DQA1*03:01P/DQB1*02:01P | 0.028 |  |  | 0.028 |  |
| DQ2 | DQA1*05:01P/DQB1*02:01P | 0.519 |  |  | 0.519 |  |
| DQ7 | DQA1*03:01P/DQB1*03:01P | 0.215 |  |  | 0.215 | 0.60 |
| DQ7 | DQA1*04:01P/DQB1*03:01P | 0.002 |  |  | 0.002 |  |
| DQ7 | DQA1*05:01P/DQB1*03:01P | 0.739 |  |  | 0.739 |  |
| DQ7 | DQA1*06:01P/DQB1*03:01P | 0.060 |  |  | 0.060 |  |
| DQ8 | DQA1*02:01P/DQB1*03:02P | 0.005 |  |  | 0.005 | 0.99 |
| DQ8 | DQA1*03:01P/DQB1*03:02P | 0.995 |  |  | 0.995 |  |
| DQ9 | DQA1*02:01P/DQB1*03:03P | 0.752 |  |  | 0.752 | 0.63 |
| DQ9 | DQA1*03:01P/DQB1*03:03P | 0.248 |  |  | 0.248 |  |
| DQ4 | DQA1*03:01P/DQB1*04:02P | 0.189 |  |  | 0.189 | 0.69 |
| DQ4 | DQA1*04:01P/DQB1*04:02P | 0.811 |  |  | 0.811 |  |
| DQ5 | DQA1*01:01P/DQB1*05:01P | 0.609 |  |  | 0.609 | 0.44 |
| DQ5 | DQA1*01:01P/DQB1*05:02P | 0.007 |  |  | 0.007 |  |
| DQ5 | DQA1*01:01P/DQB1*05:03P | 0.239 |  |  | 0.239 |  |
| DQ5 | DQA1*01:01P/DQB1*05:44P | 0.002 |  |  | 0.002 |  |
| DQ5 | DQA1*01:02P/DQB1*05:01P | 0.011 |  |  | 0.011 |  |
| DQ5 | DQA1*01:02P/DQB1*05:02P | 0.113 |  |  | 0.113 |  |
| DQ5 | DQA1*01:02P/DQB1*05:04P | 0.004 |  |  | 0.004 |  |
| DQ5 | DQA1*01:03P/DQB1*05:03P | 0.012 |  |  | 0.012 |  |
| DQ6 | DQA1*01:01P/DQB1*06:02P | 0.001 |  |  | 0.001 | 0.29 |
| DQ6 | DQA1*01:01P/DQB1*06:04P | 0.001 |  |  | 0.001 |  |
| DQ6 | DQA1*01:02P/DQB1*06:01P | 0.030 |  |  | 0.030 |  |
| DQ6 | DQA1*01:02P/DQB1*06:02P | 0.445 |  |  | 0.445 |  |
| DQ6 | DQA1*01:02P/DQB1*06:03P | 0.007 |  |  | 0.007 |  |
| DQ6 | DQA1*01:02P/DQB1*06:04P | 0.172 |  |  | 0.172 |  |
| DQ6 | DQA1*01:02P/DQB1*06:09P | 0.038 |  |  | 0.038 |  |
| DQ6 | DQA1*01:02P/DQB1*06:39P | 0.001 |  |  | 0.001 |  |
| DQ6 | DQA1*01:03P/DQB1*06:01P | 0.056 |  |  | 0.056 |  |
| DQ6 | DQA1*01:03P/DQB1*06:02P | 0.001 |  |  | 0.001 |  |
| DQ6 | DQA1*01:03P/DQB1*06:03P | 0.239 |  |  | 0.239 |  |
| DQ6 | DQA1*01:10P/DQB1*06:01P | 0.008 |  |  | 0.008 |  |

*Table S1: Frequencies of HLA-alleles for serological repeated mismatches in three regional cohorts*
For all noted serological repeated mismatches in our study, the corresponding frequencies of alleles are presented in this table as noted in three separate local cohorts. This is a cohort of renal transplant recipients who were transplanted at the LUMC from 2016 onwards and their donors, a cohort of blood donors in Leiden and its vicinity from 2008 and a cohort of registered German bone marrow donors (DKMS) from 2010 who had indicated to be of Dutch descent. Allefrequencies.net was utilized to obtain the data on the latter two cohorts. The probability of a serological repeated mismatch also being a molecular repeated mismatch is presented in the last column and is calculated based on the mean frequency of occurrence of the corresponding alleles.

**Supplemental table 2**

| **Most frequent linkage** | **DQA1/DQB1 alleles** | **DRB1 allele** |
| --- | --- | --- |
| 1st | DQA1*02:01P/DQB1*02:01P | DRB1*07:01 |
| 2nd | DQA1*02:01P/DQB1*02:01P | DRB1*13:03 |
| 1st | DQA1*03:01P/DQB1*02:01P | DRB1*09:01 |
| 2nd | DQA1*03:01P/DQB1*02:01P | DRB1*04:05 |
| 1st | DQA1*05:01P/DQB1*02:01P | DRB1*03:01 |
| 1st | DQA1*03:01P/DQB1*03:01P | DRB1*04:XX |
| 2nd | DQA1*03:01P/DQB1*03:01P | DRB1*13:01 |
| 1st | DQA1*04:01P/DQB1*03:01P | DRB1*08:04 |
| 1st | DQA1*05:01P/DQB1*03:01P | DRB1*11:XX |
| 2nd | DQA1*05:01P/DQB1*03:01P | DRB1*13:XX |
| 3rd | DQA1*05:01P/DQB1*03:01P | DRB1*12:01 |
| 1st | DQA1*06:01P/DQB1*03:01P | DRB1*12:02 |
| 1st | DQA1*03:01P/DQB1*03:02P | DRB1*04:XX |
| 1st | DQA1*02:01P/DQB1*03:03P | DRB1*07:01 |
| 1st | DQA1*03:01P/DQB1*03:03P | DRB1*09:01 |
| 1st | DQA1*03:01P/DQB1*04:02P | DRB1*04:XX |
| 1st | DQA1*04:01P/DQB1*04:02P | DRB1*08:XX |
| 2nd | DQA1*04:01P/DQB1*04:02P | DRB1*03:02 |
| 1st | DQA1*01:01P/DQB1*05:01P | DRB1*01:XX |
| 2nd | DQA1*01:01P/DQB1*05:01P | DRB1*10:01 |
| 1st | DQA1*01:01P/DQB1*05:03P | DRB1*14:XX |
| 1st | DQA1*01:02P/DQB1*05:02P | DRB1*16:XX |
| 2nd | DQA1*01:02P/DQB1*05:02P | DRB1*15:XX |
| 1st | DQA1*01:02P/DQB1*06:02P | DRB1*15:XX |
| 1st | DQA1*01:02P/DQB1*06:04P | DRB1*13:02 |
| 1st | DQA1*01:03P/DQB1*06:01P | DRB1*15:XX |
| 1st | DQA1*01:03P/DQB1*06:03P | DRB1*13:01 |

*Table S2: Observed frequently occurring linkage between DRB1 and DQA1/DQB1 in our cohort of renal transplant recipients and their donors from 2016 onwards at Leiden University Medical Centre*Linkages are presented per known DQA1/DQB1 heterodimer that have a frequency of at least more than 0.05 on their own, as shown in table S1. DRB1 alleles with second-field indicated as “XX” represent multiple alleles within the shared first-field group that were observed to be linked to the DQA1/DQB1 typing.

**Chapter III: Supplementary results**
These supplementary results detail the full transplant follow-up for graft survival (Supplemental Figures 1 & 2), Biopsy-proven rejection (Supplemental Figure 3) the differentiation of Banff’22 classified ABMR and TCMR in the Leiden cohort (Supplemental Figure 4 & 5) and the full transplant follow-up for development of DSA (Supplemental Figure 6).

**Supplemental Figure 1**


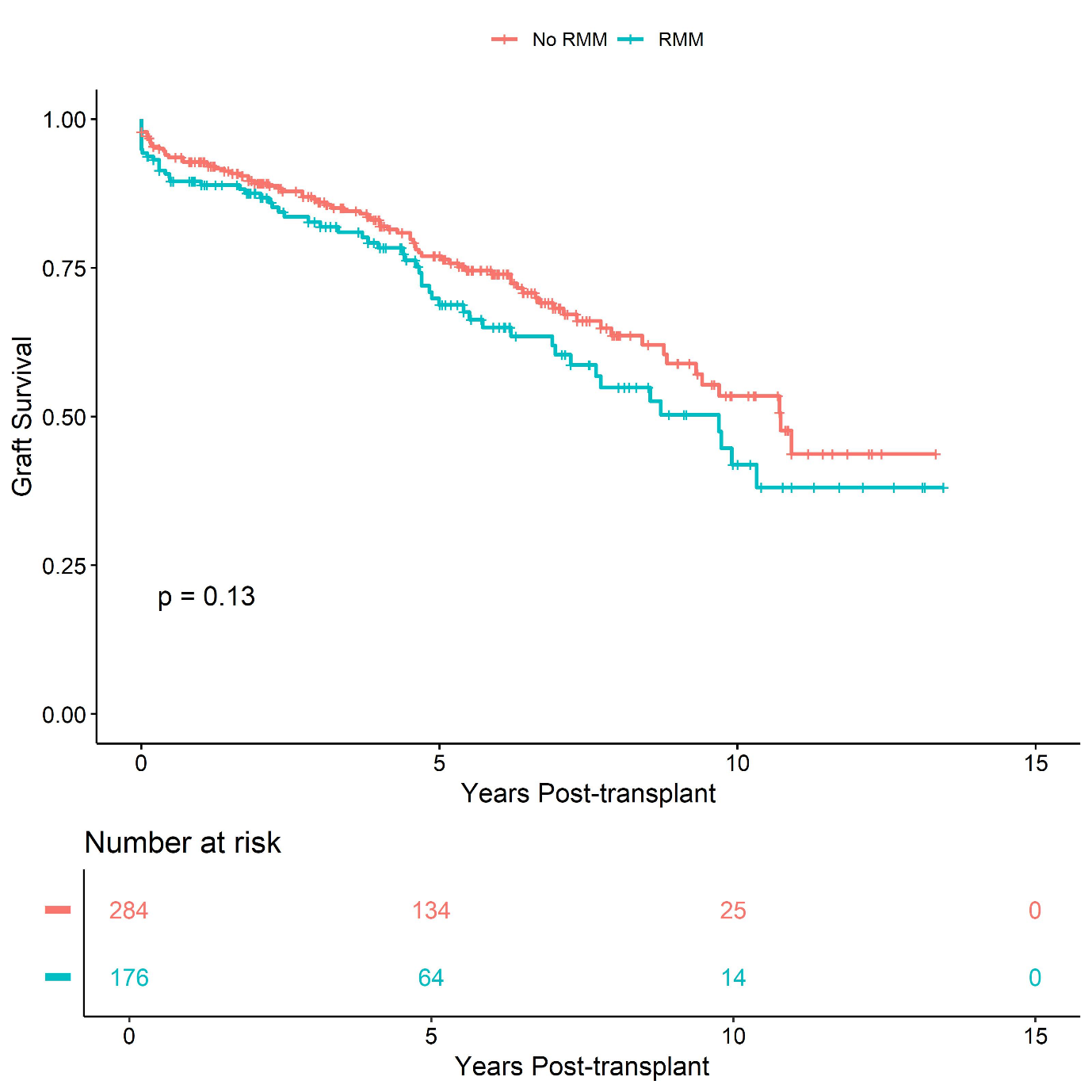


*Supplemental Figure 1: Total renal allograft survival, stratified by repeated mismatch*
All-cause graft loss is defined by either return to dialysis, pre-emptive new renal transplant, or patient death. Statistical comparison between groups is performed through the Gehan-Breslow-Wilcoxon method.
RMM: Repeated HLA-mismatch.

**Supplemental Figure 2**


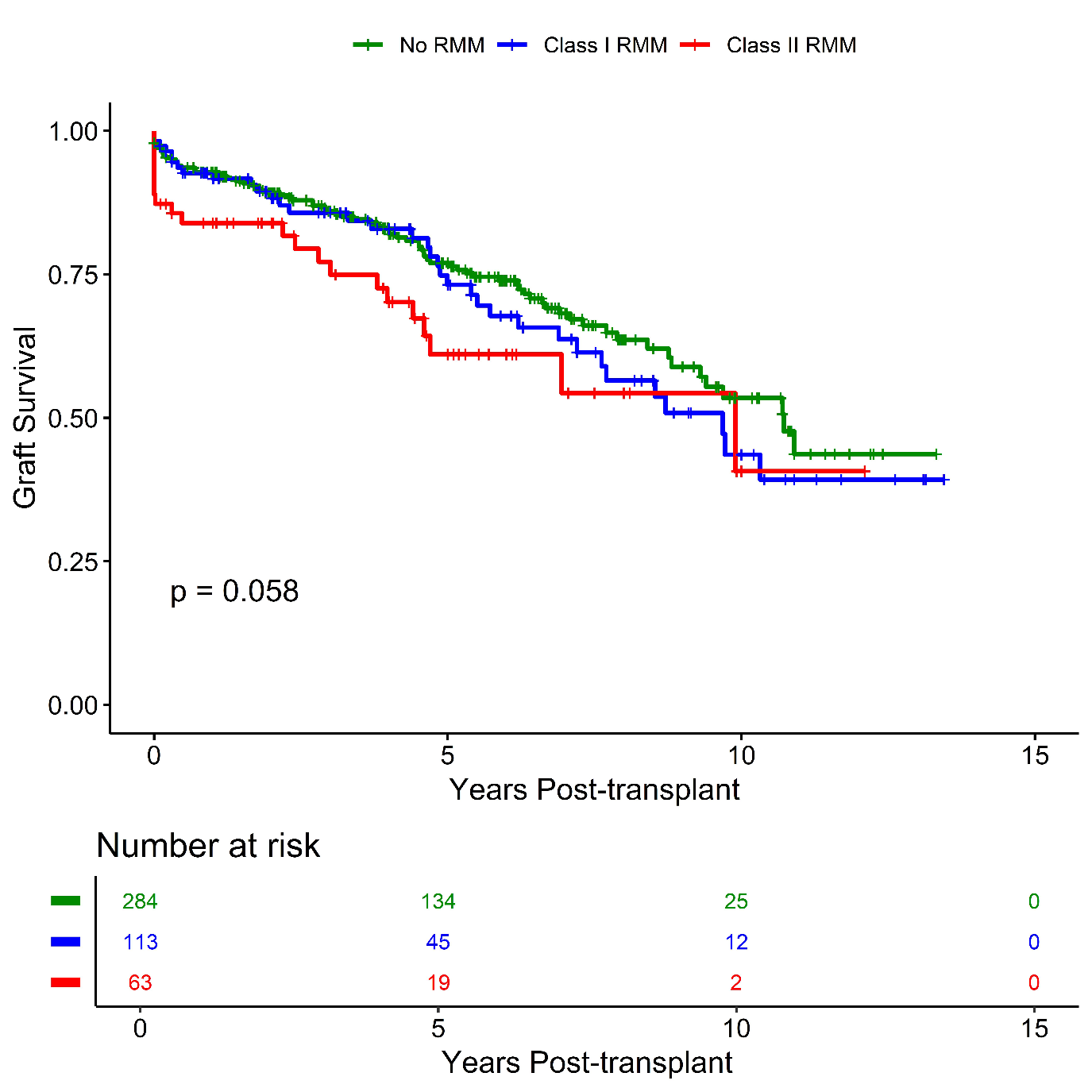


*Supplemental Figure 2: Total renal allograft survival, stratified by repeated mismatch class*
All-cause graft loss is defined by either return to dialysis, pre-emptive new renal transplant, or patient death. Statistical comparison between groups is performed through the Gehan-Breslow-Wilcoxon method.
RMM: Repeated HLA-mismatch.

**Supplemental Figure 3**

**
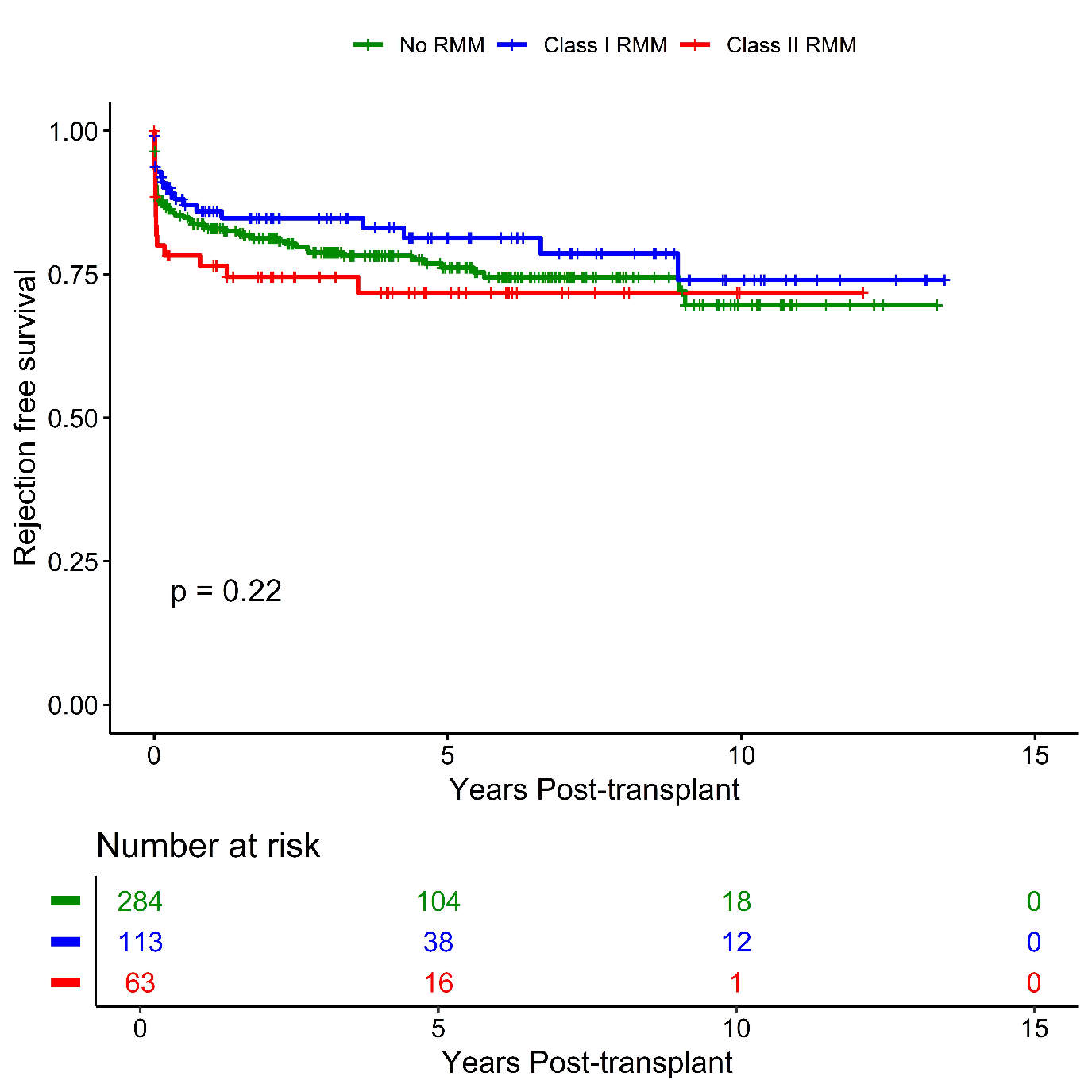
**

*Supplemental Figure 3: Total biopsy-proven rejection-free survival, stratified for repeated mismatch per HLA-class*
Biopsy-proven rejection is defined as reported by the transplant centers. Statistical comparison between groups is performed through the Gehan-Breslow-Wilcoxon method.
RMM: Repeated HLA-mismatch.

Antibody-mediated rejection
To discern whether the increased risk of biopsy-proven rejections was related to specifically ABMR or TCMR, we analyzed the effects of serological RMM on these entities separately. These analyses are restricted to LUMC patients only, considering only their biopsies were all assessed according to Banff ’22 criteria.
One- and five-year ABMR-free survival rates were 93% and 87% for patients without RMM; 96% and 80% for those with class I RMM; and 80% and 60% for those with class II RMM (Figure S4). The respective univariate ABMR hazard ratio at one- and five-years post-transplant compared to non-RMM was 0.59 ([95%CI 0.07 – 5.31, P=0.64) and 1.32 ([95%CI 0.39 – 4.52], P=0.66) for class I RMM; and 3.11 ([95%CI 0.57 – 17.0], P=0.19) and 2.97 ([95%CI 0.77 – 11.50], P=0.12) for class II RMM. The event rate for ABMR in this cohort did not allow for meaningful multivariable analysis.

T-cell mediated rejection
TCMR-free survival rates at one- and five-years post-transplant were 86% and 80% for patients without RMM, 84% and 73% for those with class I RMM, and 89% and 59% for class II RMM. There were no significant differences between RMM classes at either one- or five-years post-transplant (Figure S5) The respective univariate hazard ratio at one-year and total follow-up for TCMR compared to non-RMM was 1.14 ([95%CI 0.34 – 3.78], P=0.83) and 1.27 ([95%CI 0.47 – 3.44], P=0.64) for class I RMM; and 1.14 ([95%CI 0.34 – 3.78], P=0.27) and 1.32 ([95%CI 0.29 – 5.94], P=0.88) for class II RMM. The event rate for TCMR in this cohort did not allow for meaningful multivariable analysis.


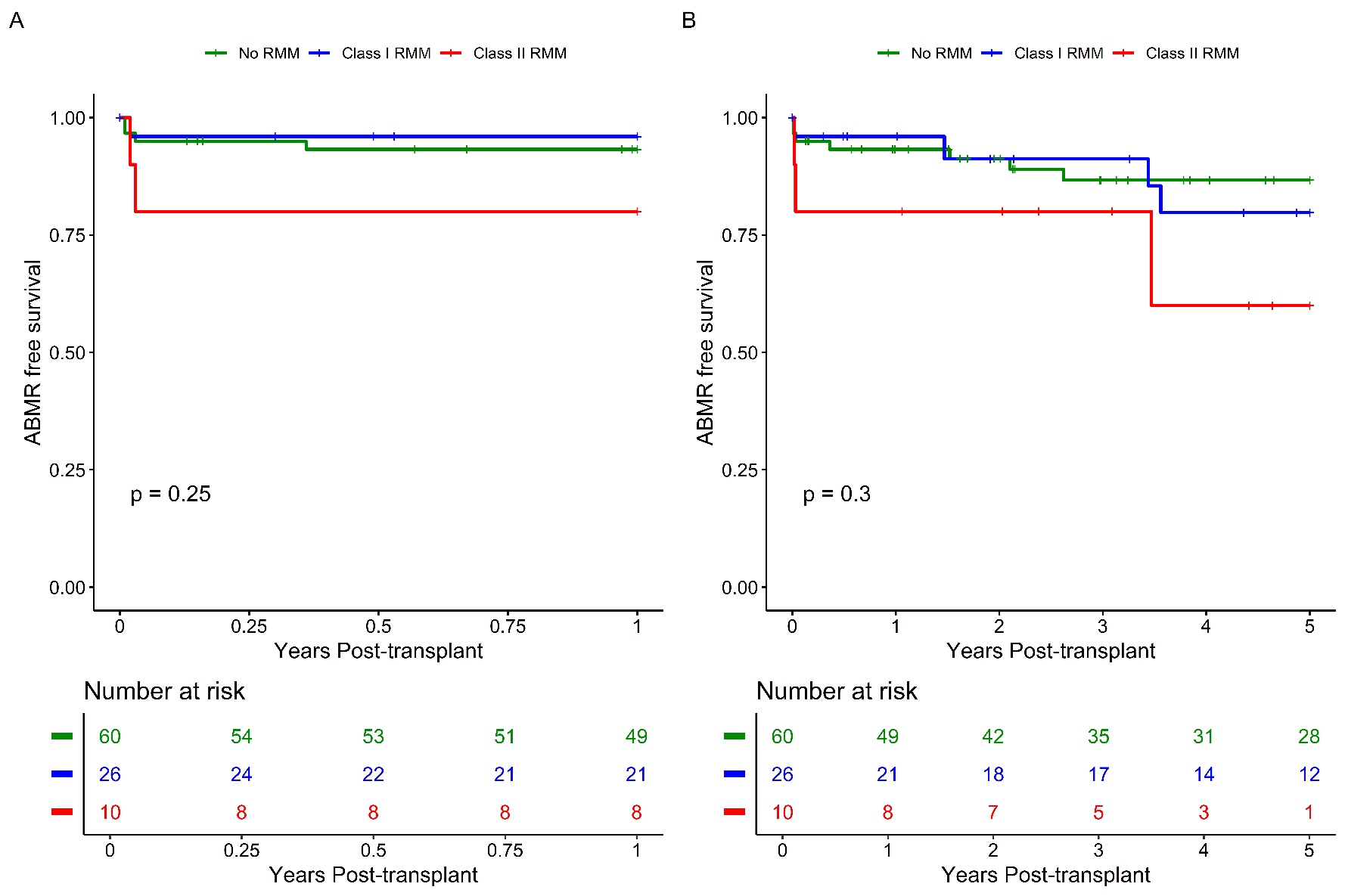
**Supplemental Figure 4**

*Supplemental Figure 4: ABMR-free survival within the LUMC cohort, stratified for repeated mismatch per HLA-class*Figure S4A: Early ABMR-free survival truncated at one-year post-transplant in the LUMC cohort. Figure S4B: ABMR-free survival truncated at five-years post-transplant in the LUMC cohort.
Statistical comparison between groups is performed through the Gehan-Breslow-Wilcoxon method.
ABMR: Antibody-mediated rejection; LUMC: Leiden University Medical Centre; RMM: Repeated HLA-mismatch.


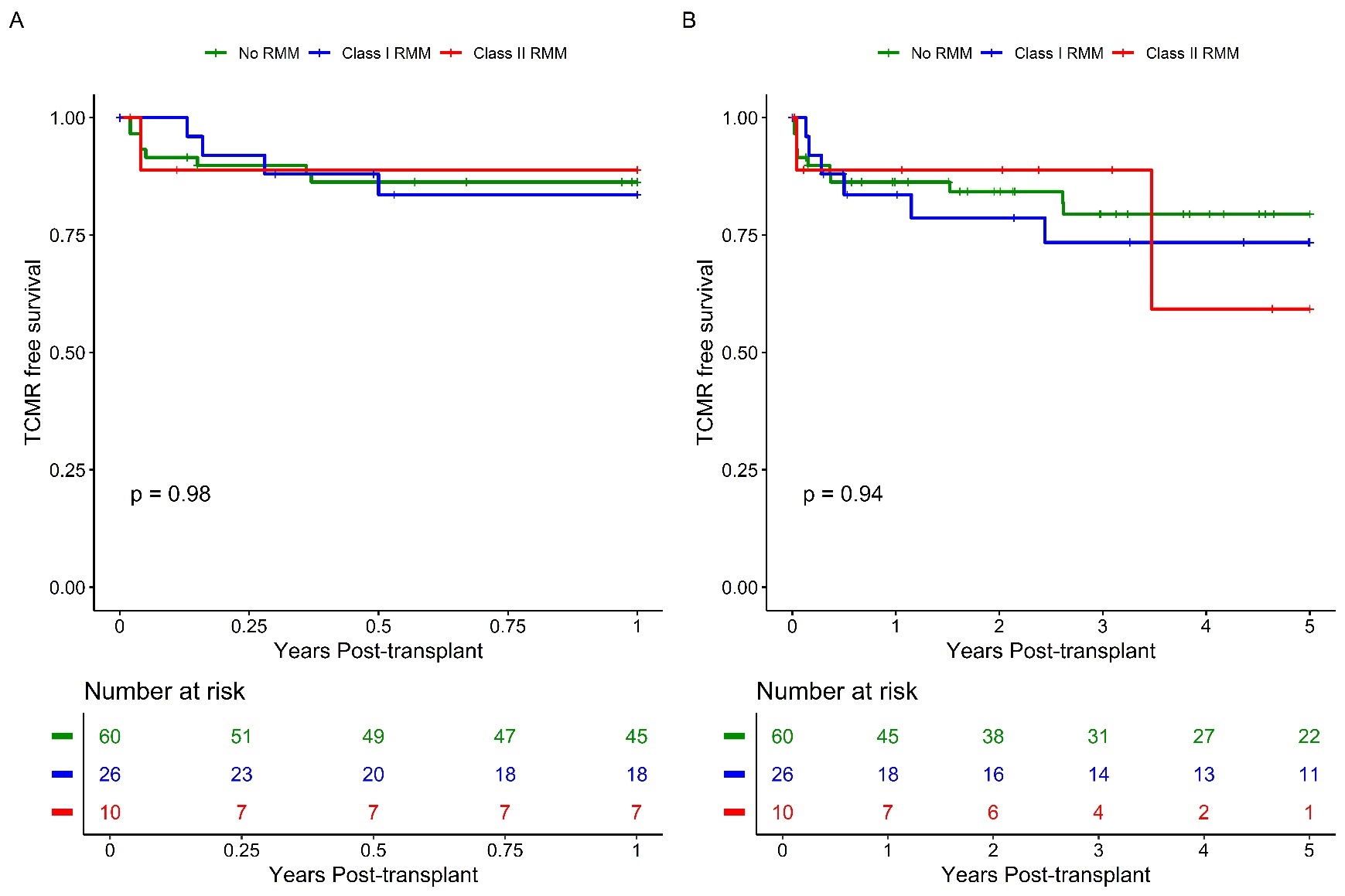
 **Supplemental Figure 5**

*Supplemental Figure 5: TCMR-free survival within the LUMC cohort, stratified by repeated mismatch class*Figure S5A: Early TCMR-free survival truncated at one-year post-transplant in the LUMC cohort. Figure S5B: TCMR-free survival truncated at five-years post-transplant in the LUMC cohort.
Statistical comparison between groups is performed through the Gehan-Breslow-Wilcoxon method.
LUMC: Leiden University Medical Centre; RMM: Repeated HLA-mismatch; TCMR: T-cell mediated rejection;

**Supplemental Figure 6**


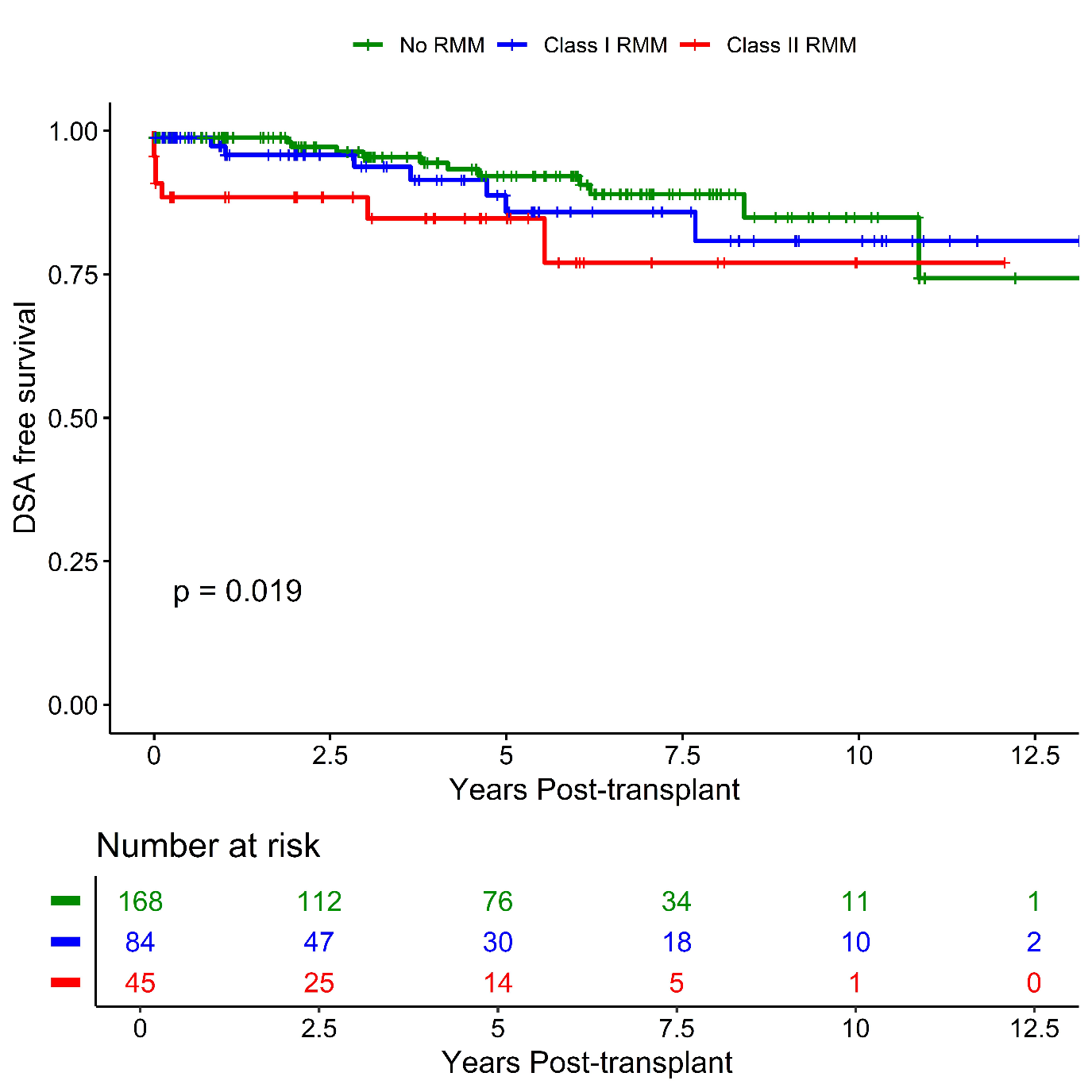


*Supplemental Figure 6: Total DSA-free survival, stratified for repeated mismatch per HLA-class*Post-transplant DSA-free survival for the LUMC and EMC centers. Post-transplant DSA data were unavailable for the UMCG center. Statistical comparison between groups is performed through Gehan-Breslow-Wilcoxon method.
RMM: Repeated HLA-mismatch.

**Chapter IV: Supplementary references**

1. Allelefrequencies.net: The Allele Frequency Net Database; 2019 [Available from: <http://allelefrequencies.net/default.asp>.
